# Rubisco kinetic acclimation at the holoenzyme level

**DOI:** 10.1073/pnas.2519914123

**Published:** 2026-04-15

**Authors:** Bryce Askey, Maddie Ceminsky, Elena Scott, Yongsheng Wang, Zhen Guo Oh, Stavros Azinas, Arthur Laganowsky, Laura Helen Gunn

**Affiliations:** ^a^Plant Biology Section, School of Integrated Plant Science, Cornell University, Ithaca, NY 14853; ^b^Department of Chemistry, Texas A&M University, College Station, TX 77843; ^c^Department of Molecular Medicine, Cornell University, Ithaca, NY 14853; ^d^Department of Biochemistry and Biophysics, Science for Life Laboratory, Stockholm University, Solna 171 65, Sweden

**Keywords:** Rubisco, CO_2_ fixation, kinetic acclimation, cryo-EM, structure–function

## Abstract

In plants, the CO_2_-fixing enzyme Rubisco is hexadecameric, with each mature holoenzyme containing eight small subunits (SSus). Many plants express multiple SSus and vary their expression in response to environmental cues. Previous work indicates that this may allow fine-tuning of Rubisco’s performance in a variable environment (i.e., kinetic acclimation). Despite SSu pools being heterogeneous and dynamic, nearly no evidence exists for holoenzyme-level heterogeneity. Here, we characterized the structural and kinetic plasticity of Rubisco. We first established that SSu-heterogeneous Rubisco exists in *Arabidopsis thaliana* and quantified the prevalence of heterogeneity. We found SSu-heterogeneous Rubisco to make up over half of the Rubisco pool when heterologously expressed. This Rubisco contained at least four unique SSu ratios, indicating a variety of holoenzyme arrangements are possible. We then tested the kinetic effect of different SSus and found heterogeneity to have an antagonistic effect on substrate and inhibitor affinity. Kinetic differences between the SSus correlated with changes in local flexibility, and cryo-EM analysis illustrated a structural mechanism through which SSus may influence catalysis. Our kinetic and structural findings align with the hypothesized role of SSus in kinetic acclimation, as we observed the warm temperature-expressed SSu of *A. thaliana* to confer a stabilizing effect to the active site relative to the cool temperature-expressed SSu. This increase in stability manifested as a reduction in flexibility and increase in substrate affinity, indicating that fine-tuning of local stability may underlie Rubisco kinetic acclimation.

## Significance

Kinetic acclimation enables proteins to adjust their activity in response to environmental perturbations. For the CO_2_-fixing enzyme Rubisco, kinetic acclimation may be conferred by its small subunits. Plants express multiple small subunits and vary their expression with temperature. Here, we demonstrate that different small subunits can bind to the same Rubisco to form a heterogeneous holoenzyme. These small subunits had distinct kinetic effects which aligned with changes in holoenzyme structure and stability. Our findings indicate that small subunits enable Rubisco kinetic acclimation via manipulation of flexibility. By assembling a more rigid active site in higher temperatures and a more flexible one in lower temperatures, plants maximize the efficiency of their Rubisco, and thus photosynthesis, over a wide range of temperatures.

Ribulose-1,5-bisphosphate (RuBP) carboxylase/oxygenase (Rubisco) functions in the Calvin–Benson–Bassham (CBB) cycle to catalyze carboxylation, the fixation of gaseous CO_2_ onto the five-carbon sugar acceptor RuBP. As its name suggests, Rubisco is bifunctional, capable of an alternative oxygenation reaction with O_2_. Oxygenation produces a dead-end by-product which must be recycled via photorespiration (1–3). Although closely intertwined with other essential pathways, photorespiration consumes energy and releases previously fixed CO_2_, with models indicating a potential 12 to 55% increase in gross photosynthesis if it were eliminated (4, 5).

The most abundant class of Rubisco, Form I, is an L_8_S_8_ hexadecamer with eight large subunits (LSus, ~50 to 55 kDa) and eight small subunits (SSus, ~12 to 18 kDa). Although SSus are necessary for the solubility and activity of all extant Form I Rubiscos, the minimal catalytic unit and hypothesized ancestral state of Rubisco is an LSu dimer (L_2_), with two active sites at the LSu–LSu interface (6, 7). Recent publications have reconstructed the SSu’s origins and characterized the kinetic effect of its acquisition (8–12). To summarize, SSu binding not only directly increased the CO_2_/O_2_ specificity (S_C/O_) of ancestral Rubisco, but also modified its accessible sequence space, enabling further S_C/O_ improvements needed to match rising atmospheric O_2_ levels (13, 14). Indeed, extant SSu-containing Form I Rubiscos have higher S_C/O_ than SSu-lacking forms (8, 15).

The mechanism by which the SSu exerts its kinetic effect is complex and multifaceted. Numerous studies have recorded the impact of point mutations and chimerizations (swapping one domain with another from a different SSu), especially at the LSu–SSu interface (16, 17). Although distant from the active site-forming LSu–LSu interface, residues at LSu–SSu interfaces are highly conserved, meaning LSus and SSus from related species are sometimes compatible and can assemble into hybrid holoenzymes with altered kinetics (16). Structural comparison of chimeric and hybrid Rubiscos to their native counterparts reveals only slight differences in LSu conformation, suggesting that the SSu’s influence may only become apparent during the conformational changes of catalysis (18, 19). SSus may exert an additional kinetic effect by serving as a reservoir for CO_2_, though support for this hypothesis comes only from molecular dynamics simulations and thus far lacks experimental validation (12, 20). Altogether, these works illustrate that SSu residues distant from Rubisco’s active sites can alter catalysis.

Plants may take advantage of the SSu’s ability to affect kinetics by modulating their expression in response to environmental cues. In contrast to the LSu which is encoded by a single gene (*rbcL*), most plants and green algae express multiple *rbcS* genes which contribute to the total SSu pool (16). The model plant *Arabidopsis thaliana* has four *rbcS* genes, encoding four SSus which can be grouped into A- and B-families. Temperature-dependent expression of *rbcS* genes results in distinct SSu pools between warm-grown (30 °C) and cold-grown (10 °C) plants (21, 22). A-family SSus make up 22% of the SSu pool in warm-grown plants but 62% in cold-grown plants, with B-family SSus following the opposite pattern. When purified from warm-grown plants, Rubisco has a lower CO_2_ turnover number (k_cat,C_), but higher CO_2_ affinity (lower K_C_) and S_C/O_ vs. Rubisco purified from cold-grown plants (22). Because higher temperatures exacerbate Rubisco’s oxygenation tendencies, temperature-dependent incorporation of a higher S_C/O_-conferring SSu may help minimize photorespiratory losses and maintain high levels of carbon fixation (5). Although other mechanisms (e.g., temperature-dependent posttranslational modification) cannot be completely ruled out, these data indicate a role for SSus in kinetic acclimation, with their differential expression potentially enabling biochemical plasticity (23, 24).

The prevalence of multigene *rbcS* families and their responsiveness to environmental cues illustrates that SSu pools in planta are heterogeneous and dynamic. Despite this, all Form I Rubisco structures available on the Protein Databank (PDB) are SSu-homogeneous, meaning every one of the eight positions on the holoenzyme is occupied by the same SSu (Dataset S1). The only previous report of Rubisco SSu heterogeneity is a now-retracted structure of spinach (*Spinacia oleracea*) Rubisco (PDB 1BUR and 1IR1) (25, 26). In this work, we tested if SSu homogeneity is a technical artifact or a true biological phenomenon. We first established the existence of SSu-heterogeneous Rubisco in planta using consecutive affinity purification. Via heterologous expression in *Escherichia coli*, we found that SSu-heterogeneous Rubisco makes up 54 to 72% of the total Rubisco pool, and that heterogeneity can be achieved through at least four unique SSu ratios. Heterologous expression also allowed us to isolate the biochemical plasticity conferred by a multigene *rbcS* family from potentially confounding differences in posttranslational modification. Finally, we solved two SSu-homogeneous structures for *A. thaliana* Rubisco and identified key differences in flexibility which may contribute to kinetic variation.

## Results

### SSu-Heterogeneous Rubisco Assembles In Planta.

**Fig. 1.**
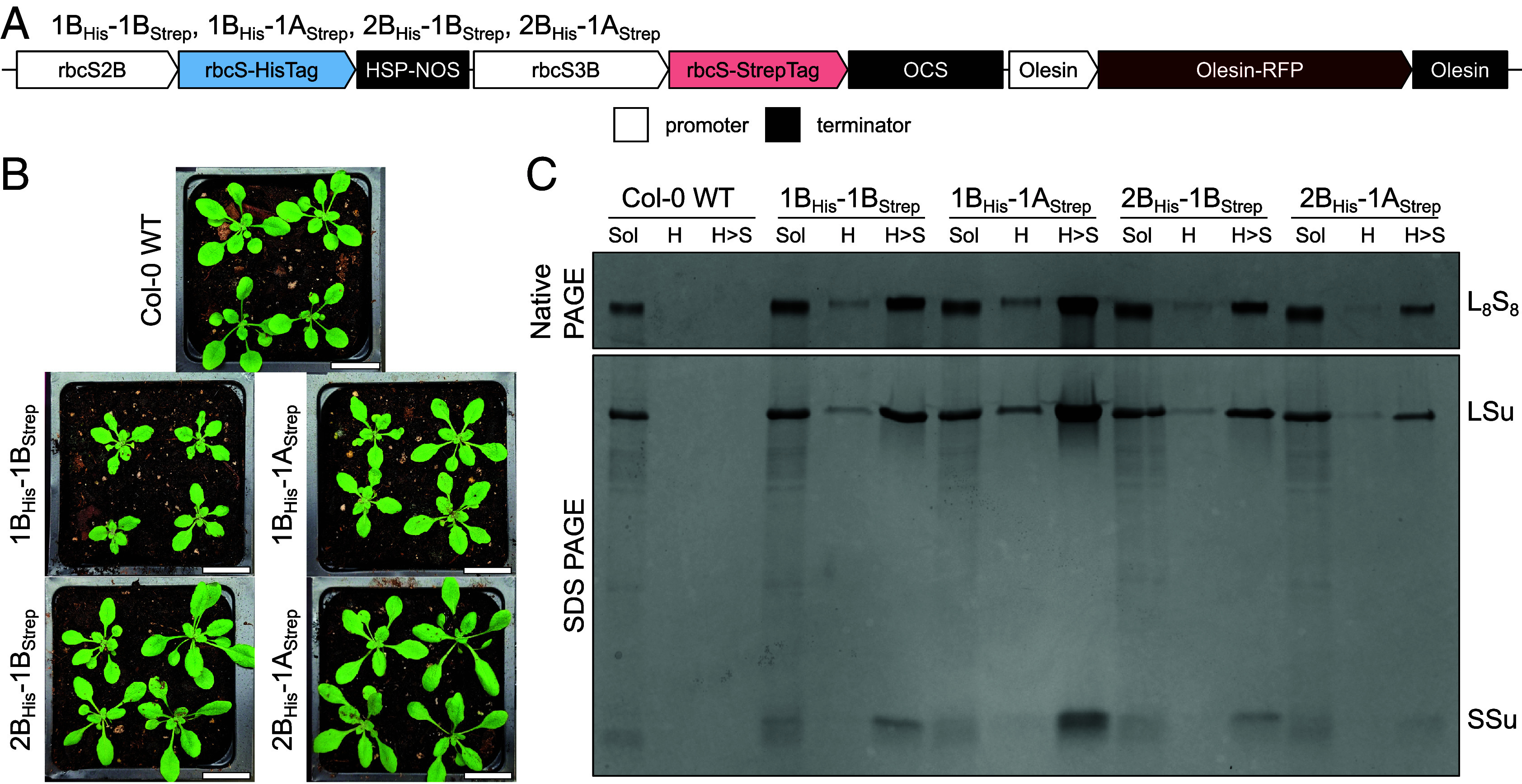
SSu-heterogeneous Rubisco can be purified from *A. thaliana* plants. (*A*) Dual-SSu construct design used to generate transformed plant lines. (*B*) 3-wk-old T2 plants. (Scale bar, 2 cm.) (*C*) Native and SDS PAGE of total soluble and affinity purified protein from T3 plants. Rubisco bands in H > S lanes appear more intense than those in H lanes because H > S fractions were concentrated prior to loading. Sol: total soluble; H: HisTrap purified; H > S: HisTrap and StrepTrap purified; RFP: red fluorescent protein.

If heterogeneity is possible in planta, simultaneous expression of His- and Strep-tagged SSus should result in assembly of Rubisco with both affinity tags. This SSu-heterogeneous Rubisco can then be isolated via consecutive His- and StrepTrap purification. We first used SSu-homogeneous Rubisco to verify that only SSu-heterogeneous Rubisco would remain after this consecutive purification (*SI Appendix*, Fig. S1) (27–29). We then transformed *A. thaliana* Col-0 to express pairs of His-tagged (e.g., SSu2B_His_) and Strep-tagged (e.g., SSu1A_Strep_) SSus, with expression driven by the *rbcS2B* and *rbcS3B* promoters. Because two (SSu2B and SSu3B) of *A. thaliana*’s four SSus are identical at the mature protein level, we selected just the *rbcS2B* ORF to limit the number of possible pairwise combinations (*SI Appendix*, Fig. S2). We generated three experimental lines (1B_His_-1A_Strep_, 2B_His_-1B_Strep_, and 2B_His_-1A_Strep_), and a control line (1B_His_-1B_Strep_) which we used to verify that the tags did not interfere with compatibility (Fig. 1*A* and Dataset S2).

After transforming plants, we used western blotting to select T1 lines with high transgene expression, and the pFAST-R selection marker to establish homozygosity in the T2 generation (*SI Appendix*, Fig. S3) (30). Three-week-old T2 1B_His_-1B_Strep_ and 1B_His_-1A_Strep_ plants showed visible growth stunting relative to wild type (WT), but 2B_His_-1B_Strep_ and 2B_His_-1A_Strep_ plants appeared WT-like (Fig. 1*B*). We verified transgene presence in these T2 plants (*SI Appendix*, Fig. S4 and Table S1), and from 4-wk-old T3 plants, collected total soluble protein (Sol), protein surviving HisTrap purification (H), and protein surviving successive His- and StrepTrap purification (H > S). As expected, no Rubisco from WT plants survived the first step of HisTrap purification. In contrast, some Rubisco from all transformant lines survived successive HisTrap and StrepTrap purification, indicating that every pairwise combination of SSus is compatible and capable of heterogeneity (Fig. 1*C*).

### Heterologously Expressed Rubisco Is 54 to 72% SSu-Heterogeneous.

**Fig. 2.**
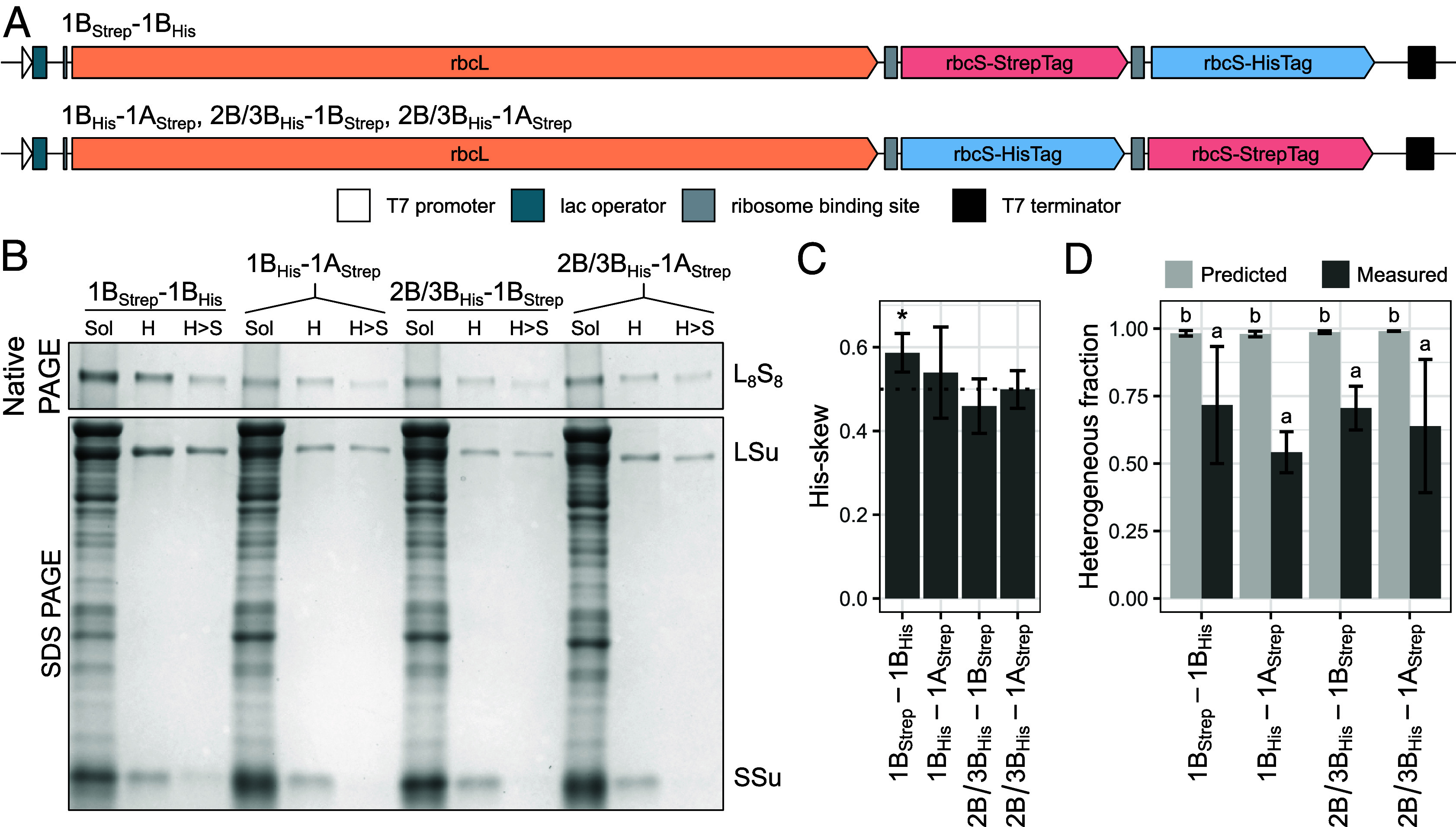
Heterologous expression enables quantification of heterogeneity. (*A*) Dual-SSu construct designs used for heterologous expression experiments. (*B*) Representative native and SDS PAGE of total soluble and affinity-purified protein from dual SSu-expressing *E. coli*. (*C*) His-skew varies between constructs (n = 6). His-skew quantifies the evenness of His- and Strep-tagged SSu incorporation, and is calculated by dividing the amount of initial Rubisco that is His-tagged by the total amount of initial Rubisco. The dotted line indicates His-skew = 0.5, which occurs when both SSus are incorporated with equal frequency. Asterisks “*” indicate values which significantly differ from 0.5, as determined with a one-sample *t* test with significance cutoff 0.05. (*D*) Predicted heterogeneity exceeds measured heterogeneity across all pairwise combinations of SSus (n = 6). Predicted heterogeneity is calculated with a His-skew-informed binomial distribution which assumes that the binding of each SSu to a holoenzyme is an independent event. Measured heterogeneity is calculated by dividing the amount of Rubisco remaining after successive purification by the amount of input Rubisco. Error bars indicate SD. Letters indicate ranking of means following one-way ANOVA and post hoc Tukey HSD test with significance cutoff 0.05. Sol: total soluble; H: HisTrap purified; H > S: HisTrap and StrepTrap purified.

Quantifying the prevalence of heterogeneity is confounded by the presence of untagged SSus. We instead adapted a published method for Rubisco expression in BL21(DE3) *E. coli* by adding a second Strep-tagged SSu to the pRSF plasmid (Fig. 2*A* and Dataset S2) (27–29). As in planta, we generated three experimental lines (1B_His_-1A_Strep_, 2B/3B_His_-1B_Strep_, and 2B/3B_His_-1A_Strep_) and one control line (1B_Strep_-1B_His_). Our use of “SSu2B/3B” in naming reflects the equivalence of SSu2B and SSu3B during heterologous expression. We collected soluble protein, applied consecutive His- and StrepTrap purification, and again found every pairwise combination of SSus to be capable of heterogeneity (Fig. 2*B*).

Since affinity purification is not perfectly efficient, some Rubisco will be lost even if it contains an SSu with the appropriate tag. To accurately estimate heterogeneity, we tracked the amount of Rubisco at each stage of purification by western blotting with His- and StrepTag antibodies (*SI Appendix*, Fig. S5 and Dataset S3). This allowed us to account for column efficiency and calculate corrected amounts of His- and Strep-tagged Rubisco. With a simulated dataset, we found that our correction was accurate under most conditions, but underestimated heterogeneity when column efficiency varied with the number of appropriate tags present (*SI Appendix*, Fig. S6).

Despite the near analogous construction of all Rubisco expression plasmids, we observed differences in the evenness of assembly of the two SSus between constructs (Fig. 2*C*). We refer to this evenness as His-skew and quantify it as the fraction of total L_8_S_8_-incorporated SSu which is His-tagged. His-skew for 1B_Strep_-1B_His_ was significantly greater than 0.5, meaning the His-tagged SSu was more prevalent in the initial holoenzyme pool than the Strep-tagged SSu. Differences in His-skew matter because even assembly of both SSus (His-skew = 0.5) maximizes the likelihood of SSu-heterogeneous Rubisco formation. Assuming consecutive binding of each SSu to a holoenzyme is an independent event, the likelihood of homo- and heterogeneous Rubisco formation can be modeled by a binomial distribution with n = 8 and p = His-skew. This binomial distribution predicted the fraction of SSu-heterogeneous Rubisco to range from 0.980 ± 0.010 (1B_His_-1A_Strep_) to 0.991 ± 0.001 (2B/3B_His_-1A_Strep_), meaning SSu-homogeneous Rubisco was predicted to make up no more than 2% of the total Rubisco pool (Fig. 2*D*).

We measured heterogeneity by dividing the amount of Rubisco remaining after consecutive purification by the input amount. Across all pairwise combinations, measured heterogeneity ranged from 0.542 ± 0.076 (1B_His_-1A_Strep_) to 0.717 ± 0.217 (1B_Strep_-1B_His_) (Fig. 2*D*). As demonstrated with our simulated dataset, this discrepancy between measured and predicted values could be explained by our method’s inability to fully account for variance in column efficiency with the number of affinity tags per holoenzyme. However, this error should be largely addressed by our pooling of all Rubisco-containing fractions after each purification step. Therefore, the significant difference between measured and predicted heterogeneity indicates that consecutive binding of SSus to a holoenzyme are likely not independent events, and suggests some preference for homogeneity and/or for a specific ratio of SSus in heterogeneous Rubisco.

### SSu-Heterogeneous Rubisco Can Accommodate at Least Four Unique SSu Ratios.

Measured percent heterogeneity being lower than predicted could reflect a requirement for a specific spatial arrangement of different SSus on the holoenzyme. If only some arrangements are possible, consecutive binding of SSus are not independent events and heterogeneity will not be modeled correctly by a binomial distribution. Although probing spatial arrangement via structural characterization would be ideal, the lack of SSu-heterogeneous Rubisco structures made us wary. Instead, we tested the simpler question of which SSu ratios were possible.

**Fig. 3.**
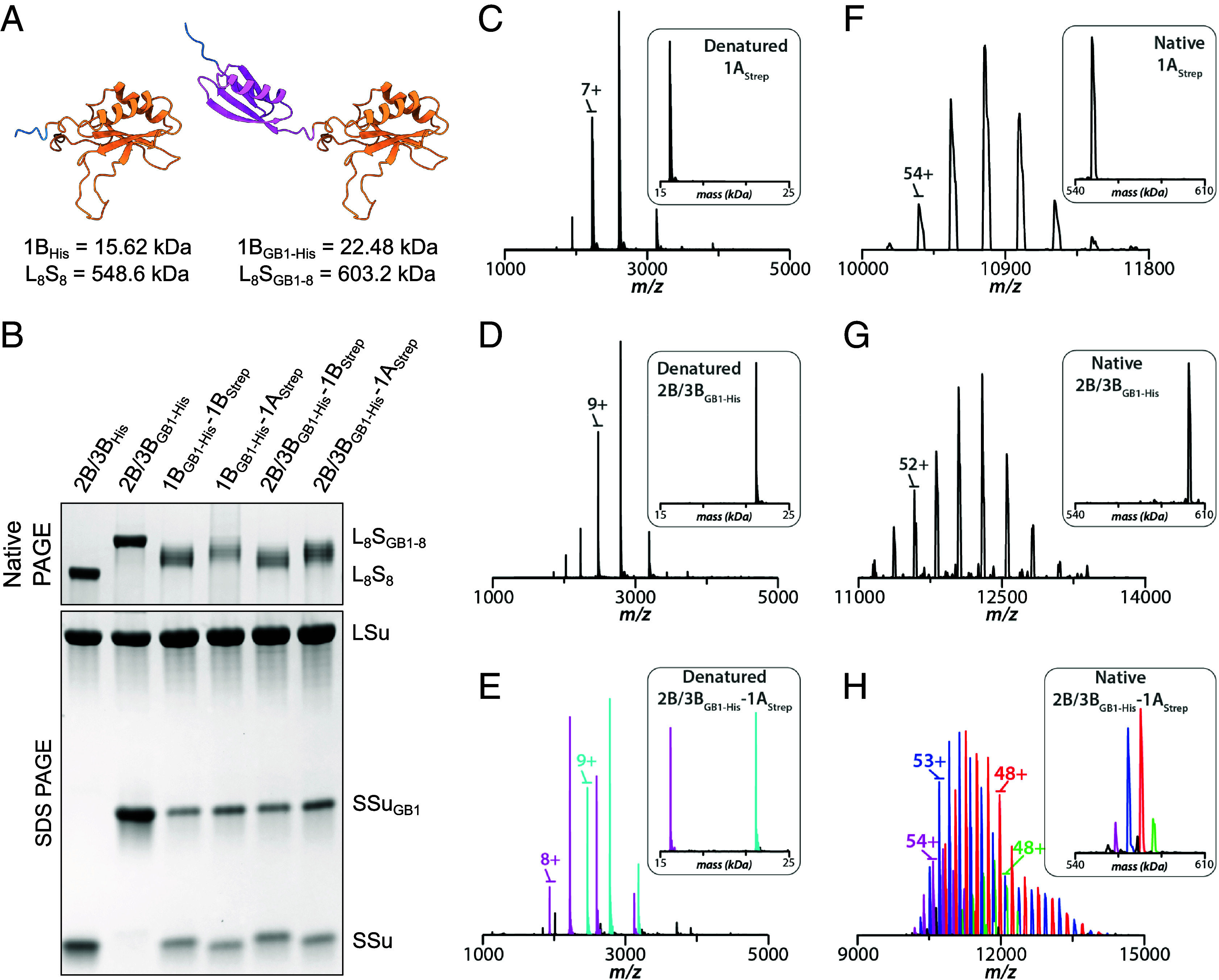
At least four different SSu stoichiometries are possible in SSu-heterogeneous Rubisco. (*A*) Comparison of 1B_His_ and Alphafold2-predicted 1B_His-GB1_ SSu structures illustrate how the addition of a GB1 tag increases the SSu’s mass (33). 2B/3B SSu in beige, HisTag in blue, GB1 tag in violet. (*B*) Native and SDS PAGE of purified Rubisco with GB1-tagged SSu. SSu-heterogeneous complexes migrate intermediate to L_8_S_8_ and L_8_S_GB1-8_ bands. MS spectra of denatured (*C*) 1A_Strep_, (*D*) 2B/3B_GB1-His_, and (*E*) 2B/3B_GB1-His_-1A_Strep_ Rubisco confirms the presence of both 1A_Strep_ and 2B/3B_GB1-His_ SSus in 2B/3B_GB1-His_-1A_Strep_ Rubisco. Deconvoluted spectra shown in *Inset*. MS spectra of native (*F*) 1A_Strep_, (*G*) 2B/3B_GB1-His_, and (*H*) 2B/3B_GB1-His_-1A_Strep_ Rubisco reveals multiple possibilities for holoenzyme-level heterogeneity. Homogeneous 1A_Strep_ and 2B/3B_GB1-His_ native spectra resolve to single peaks following deconvolution, but heterogeneous 2B/3B_GB1-His_-1A_Strep_ resolves to four peaks, reflecting four different SSu ratios.

We first analyzed Rubisco purified from WT *A. thaliana* plants and 2B/3B_His_-1A_Strep_ Rubisco expressed in *E. coli*, but were unable to resolve distinct SSu ratios (*SI Appendix*, Fig. S7). N-terminal methionine processing of the LSu has been previously observed in planta and in *E. coli*, so we hypothesized that varying degrees of processing occluded the small differences in mass between the SSus (27, 28, 31). To address this, we modified our four *E. coli* dual SSu constructs by adding the B1 domain of *Streptococcal* protein G (GB1) to the C terminus of one of the SSus (1B_GB1-His_-1B_Strep_, 1B_GB1-His_-1A_Strep_, 2B/3B_GB1-His_-1B_Strep_, and 2B/3B_GB1-His_-1A_Strep_; Dataset S2) (32). The GB1 tag is typically used to increase solubility of heterologously expressed proteins. Here, we used it to increase the mass of an SSu from 15.62 kDa to 22.48 kDa, exaggerating the stepwise difference in mass between holoenzymes with differing SSu ratios (Fig. 3*A*). We also generated a single SSu construct (2B/3B_GB1-His_) to verify that the GB1 tag does not block assembly, and to serve as an upper bound for mass when analyzing SSu-heterogeneous Rubisco.

Following purification, we used SDS PAGE to confirm that SSu-heterogeneous Rubisco contained both GB1-tagged and non-GB1-tagged SSus. On native PAGE, this heterogeneous Rubisco ran between the homogeneous 2B/3B_His_ and 2B/3B_GB1-His_ bands, reflecting its intermediate mass (Fig. 3*B*). Furthermore, the heterogeneous Rubisco appeared to separate into several bands, indicating that multiple SSu ratios are possible for all pairwise SSu combinations (Fig. 3*B*). We identified which ratios these were with mass spectrometry (MS) of denatured and native Rubisco (*SI Appendix*, Table S2).

MS of denatured Rubisco confirmed that 2B/3B_GB1-His_-1A_Strep_ Rubisco contained two SSu populations with masses matching those of homogeneous 1A_Strep_ and 2B/3B_GB1-His_ Rubisco (Fig. 3 *C*–*E*). MS of native 1A_Strep_ and 2B/3B_GB1-His_ Rubisco showed these samples were homogeneous, with singular masses of 548.08 and 602.09 kDa, respectively (Fig. 3 *F* and *G*). In contrast, 2B/3B_GB1-His_-1A_Strep_ Rubisco was heterogeneous with masses of 561.63, 586.39, 575.16, and 581.91 kDa (Fig. 3*H*). After matching measured and theoretical masses, we concluded that the following ratios of SSu1A_Strep_:SSu2B/3B_GB1-His_ were present: 6:2, 5:3, 4:4, and 3:5 (*SI Appendix*, Table S3).

### SSu-Heterogeneous Rubisco Has Decreased RuBP Affinity.

Previously, Cavanagh et al. (22) found the composition of the SSu pool to be temperature dependent, with SSu1A making up ~65% of the SSu pool in cold-grown *A. thaliana* plants, and the B-family of SSus making up ~65% in warm-grown plants. Rubisco from cold-grown plants was faster (higher k_cat,C_) but had lower affinity for CO_2_ (higher K_C_, lower S_C/O_) than Rubisco from warm-grown plants. Here, we isolate the contributions of SSu-homogeneous and heterogeneous Rubisco with affinity-tagged Rubisco purified from *E. coli*, which has previously been shown to have comparable kinetics to leaf-purified untagged Rubisco (28, 29).

**Fig. 4.**
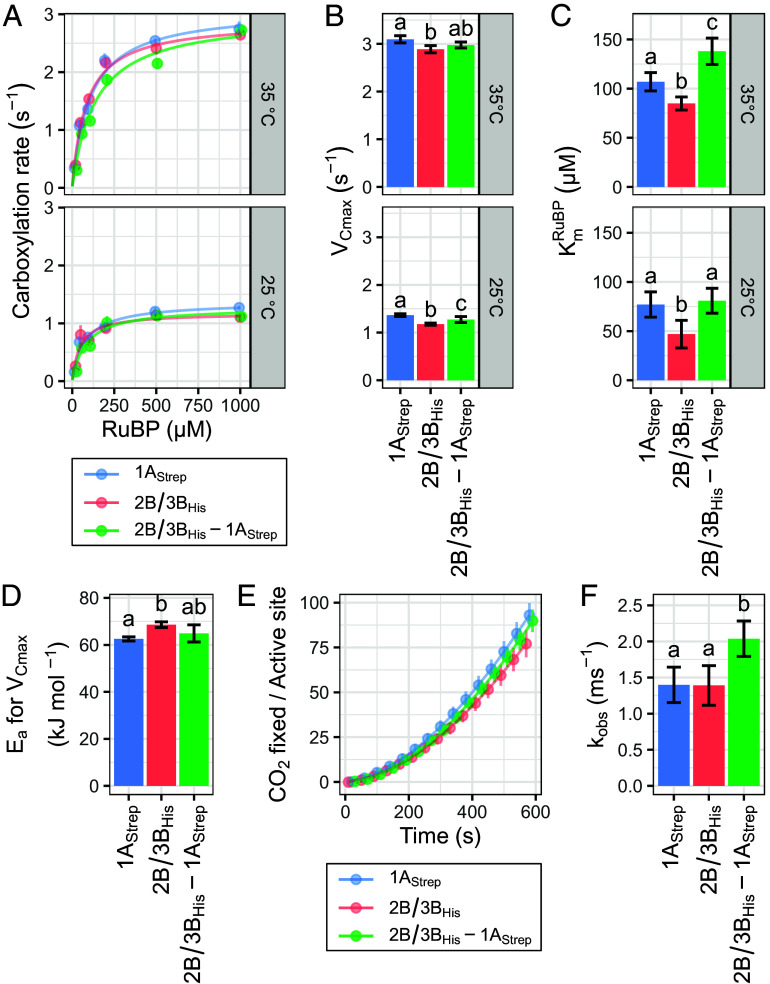
SSu-heterogeneous Rubisco has intermediate speed but lower affinity for both substrate and inhibitory RuBP. (*A*) Carboxylation rates of 1A_Strep_, 2B/3B_His_, and 2B/3B_His_-1A_Strep_ Rubisco over six RuBP concentrations at 25 and 35 °C (n = 5). A Michaelis–Menten model was fit to each replicate and the average model plotted as a line. Mean (*B*) V_Cmax_ and (*C*) K_m_^RuBP^ of fitted Michaelis–Menten models. (*D*) Mean E_a_, calculated from the difference in V_Cmax_ between 25 and 35 °C. (*E*) Activity recovery of RuBP-inhibited 2B/3B_His_, 1A_Strep_, and 2B/3B_His_-1A_Strep_ Rubisco over 10 min at 25 °C (n = 5). A rate equation for slow binding inhibition was fit to each replicate and the average model plotted as a line. (*F*) Mean k_obs_ of fitted activity recovery models. Error bars indicate SD. Letters indicate ranking of means following one-way ANOVA and post hoc Tukey HSD test with significance cutoff 0.05.

Using an NADH-coupled spectrophotometric assay, we measured the activity of purified, activated Rubisco (ECM) at six RuBP concentrations and two temperatures (Fig. 4*A* and Dataset S4) (34, 35). Matching the results of Cavanagh et al. (22), we found 1A_Strep_ to have a higher maximum carboxylation rate (V_Cmax_) than 2B/3B_His_ by a small, but significant margin which increased slightly from 25 to 35 °C (Fig. 4*B* and *SI Appendix*, Table S4). We verified this difference in carboxylation rate (V_C_) at 25 °C with the highest RuBP concentration over three biological replicates (*SI Appendix*, Fig. S8 and Table S5). SSu-heterogeneous 2B/3B_His_-1A_Strep_ exhibited a V_Cmax_ intermediate to its pure counterparts. Concerning substrate affinity (K_m_^RuBP^),1A_Strep_ Rubisco had lower affinity (higher K_m_^RuBP^) than 2B/3B_His_ (Fig. 4*C* and *SI Appendix*, Table S4). Interestingly, 2B/3B_His_-1A_Strep_ had an elevated K_m_^RuBP^ that matched 1A_Strep_ at 25 °C and exceeded both 2B/3B_His_ and 1A_Strep_ at 35 °C. Altogether, these results indicate heterogeneity has a linear effect on V_Cmax_, but an antagonistic one on K_m_^RuBP^.

We then calculated activation energy (E_a_) from our V_Cmax_ measurements at 25 and 35 °C, and found 1A_Strep_ to have a lower E_a_ for V_Cmax_ than 2B/3B_His_ (Fig. 4*D* and *SI Appendix*, Table S6). Like V_Cmax_, 2B/3B_His_-1A_Strep_ had intermediate E_a_ for V_Cmax_.

For Rubisco to catalyze its carboxylation reaction, its catalytic site must first be activated by binding of a nonsubstrate CO_2_ and an Mg^2+^ ion (36). If RuBP binds prior to activation, an inhibited complex is formed with no carboxylation activity. To follow up on our observation of differing substrate RuBP affinity, we prepared RuBP-inhibited Rubisco (ER) and measured its rate of reactivation at 25 °C (Fig. 4*E* and Dataset S4) (37, 38). We found 2B/3B_His_-1A_Strep_ Rubisco to have a higher first-order rate constant for inhibitor release (k_obs_) than its pure counterparts (Fig. 4*F* and *SI Appendix*, Table S7). Overall, our data reinforce the finding from Cavanagh et al. (22) that distinct kinetics are conferred by A- and B-family SSus, and also demonstrates that SSu-heterogeneous Rubisco has reduced affinity for substrate and inhibitory RuBP.

### SSu1A and SSu2B/3B Have Distinct Effects on Holoenzyme Stability.

**Fig. 5.**
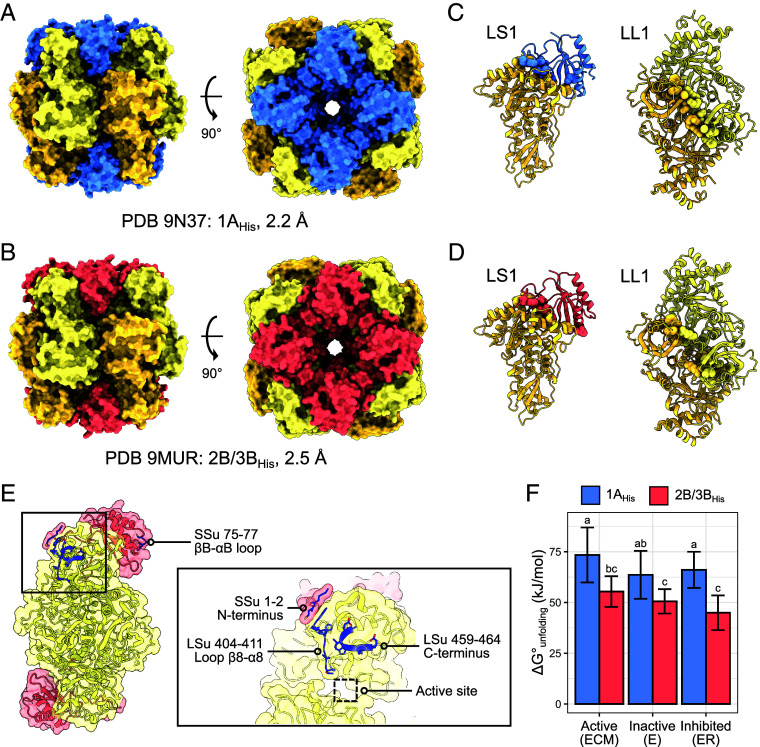
SSu1A_His_ increases the flexibility of Rubisco’s active site relative to SSu2B/3B_His_. Cryo-EM structures of heterologously-expressed Rubisco homogeneous for (*A*) SSu1A_His_ and (*B*) SSu2B/3B_His_. LSu in yellow, SSu1A_His_ in blue, SSu2B/3B_His_ in red. (*C*) L_1_S_1_ and L_2_ units from 1A_His_ structure with residues involved in hydrogen bonding at LL1 and LS1 interfaces shown as spheres. (*D*) L_1_S_1_ and L_2_ units from 2B/3B_His_ structure with residues involved in hydrogen bonding at LL1 and LS1 interfaces shown as spheres. (*E*) An L_2_S_2_ unit from the 2B/3B_His_ structure. Loops visible in the 2B/3B_His_ structure but not the 1A_His_ structure are shown in blue. The *Inset* shows an enlarged view of the boxed region. (*F*) Gibbs free energy of unfolding (ΔG°_unfolding_) of 1A_His_ and 2B/3B_His_ Rubisco in active, inactive, and RuBP-inhibited states (n = 16). Error bars indicate SD. Letters indicate ranking of means following one-way ANOVA and post hoc Tukey HSD test with significance cutoff 0.05.

To probe the biochemical mechanisms underlying observed kinetic differences, we compared cryo-EM structures of SSu-homogeneous 1A_His_ (PDB 9N37 (39)) and 2B/3B_His_ (PDB 9MUR (40)) Rubisco (Fig. 5 *A* and *B*). We collected these structures in the absence of substrate mimics (e.g., 2-carboxy-D-arabinitol 1,5-bisphosphate [CABP]), enabling us to observe differences between the holoenzymes in their more flexible “open” state. We applied similar parameters to collect and solve these structures (*SI Appendix*, Figs. S9 and S10 and Table S8). The largest difference was that Mg^2+^ was added to 1A_His_ but not 2B/3B_His_ Rubisco prior to collection, though Mg^2+^ has previously been demonstrated to have little effect on Rubisco’s secondary structure (41, 42). As expected, our structures shared a high degree of similarity (*SI Appendix*, Table S9). However, closer investigation revealed important differences.

The LS1 interface (see *SI Appendix*, Fig. S11 for interface labeling) in the 1A_His_ structure was 8.1% smaller than in the 2B/3B_His_ structure, with 7.8% fewer residues involved. (Fig. 5 *C* and *D* and *SI Appendix*, Table S10). This corresponded to 13.1% fewer nonbonded contacts and 21.2% fewer hydrogen bonds. The LL1 interface showed a similar trend, with 3.6% less area, 4.4% fewer residues, and 6.3% fewer nonbonded contacts in the 1A_His_ structure. However, we observed 24% more hydrogen bonds at the LL1 interface in our 1A_His_ structure.

We also observed differences in flexibility between the 1A_His_ and 2B/3B_His_ structures. Our map for the 2B/3B_His_ structure contained density in several SSu and LSu regions missing in the 1A_His_ structure: SSu loop βB-αB, the SSu N terminus, LSu loop β8-α8, and the LSu C terminus (Fig. 5*E* and *SI Appendix*, Fig. S2). The latter three regions are near the active site, forming extensive interactions in the 2B/3B_His_ structure. To examine if this increase in 1A_His_ local flexibility was accompanied by an increase in global flexibility, we used a thermal shift assay to evaluate the thermal stability of ECM, inactive (E), and ER 1A_His_ and 2B/3B_His_ Rubisco. Both exhibited similar melting temperatures as ECM and E which increased when inhibited (*SI Appendix*, Fig. S12, Table S11). Unexpectedly, 1A_His_ Rubisco had significantly higher Gibbs free energy of unfolding (ΔG°_unfolding_) than 2B/3B_His_, indicating that more energy is required to totally denature 1A_His_ Rubisco than 2B/3B_His_ (Fig. 5*F* and *SI Appendix*, Table S11). Greater hydrogen bonding at the LL1 and LS2 interfaces in the 1A_His_ structure may have contributed to this difference (*SI Appendix*, Table S10).

## Discussion

### Reconciling Measured Heterogeneity with the Lack of SSu-Heterogeneous Structures.

SSu-heterogeneous Rubisco exists in planta and could make up at least 54.2% of the Rubisco pool during heterologous expression (Figs. 1*C* and 2*D*). These data seemingly contradict the apparent homogeneity in all Form I Rubisco structures currently uploaded to PDB (Dataset S1). The only previously reported heterogeneity is a crystal structure for spinach Rubisco containing a 4:4 ratio of two different SSus (25). This structure was originally uploaded to PDB with ID 1BUR. However, after further refinement, the authors found that a different SSu sequence better fit their density, and republished the now SSu-homogeneous structure under ID 1IR1 (26). None of the other eleven structures for spinach Rubisco available on PDB show any heterogeneity (Dataset S1).

A crystal structure of *A. thaliana* Rubisco homogeneous for SSu1B is of particular interest when considering Rubisco SSu heterogeneity (PDB 5IU0) (43). The plants from which Rubisco was purified to yield this structure were grown at 20 °C, a temperature at which protein accumulation of A- and B-family SSus is approximately equal and *rbcS1B* represents just 8% of the total *rbcS* transcript pool (22, 44). Valegård et al. (43) hypothesized that the crystallization process may have “titrated out” Rubisco homogeneous for SSu1B, resulting in the recovery of a homogeneous structure. The differences we observed between our 1A_His_ and 2B/3B_His_ structures are consistent with this hypothesis (Fig. 5 *A*–*E*). Structural changes induced by binding of different SSus likely prevent SSu-heterogeneous Rubisco from forming tightly packed and well-ordered crystals. Our structural and thermostability data also indicate that SSu2B/3B has a local stabilizing effect relative to SSu1A (Fig. 5 *E* and *F*). Sequence-wise, mature SSu1B is more alike SSu2B/3B than SSu1A, and thus SSu1B may similarly enhance active site stability, making its crystallization more favorable (*SI Appendix*, Fig. S2) (43). As cryo-EM circumvents the need for crystallization, cryo-EM-derived structures likely better reflect the true composition of Rubisco pools in planta. However, the potential of cryo-EM to capture in planta heterogeneity remains yet to be realized, as nearly all current cryo-EM structures of Rubisco are of heterologously-expressed protein (Dataset S1).

Chloroplast import may also contribute to differences in heterogeneity in planta vs. during heterologous expression. In planta, *rbcS* genes are nuclear-encoded, and following translation in the cytosol, SSu preprotein is transported into the chloroplast via the TOC–TIC complex (45). SSu protein abundance in the chloroplast likely differs from *rbcS* transcript abundance in part due to this import. More specifically, variability in transit peptide sequence between *A. thaliana*’s SSus may influence their import efficiency by TOC–TIC, thereby altering their availability for assembly into L_8_S_8_ Rubisco (46). Preferential import of one SSu preprotein over another could increase the likelihood of assembling Rubisco homogeneous for that SSu. Overall, we emphasize that the heterogeneity we measure via heterologous expression may not match levels in planta, and that more work is needed to understand the effect of transit peptide differences on SSu preprotein import.

### SSu-Induced Differences in Structure, Function, and Stability.

Our cryo-EM structures of 1A_His_ and 2B/3B_His_ Rubisco enabled us to make a structural comparison of Rubisco with different, but high sequence identity SSu isoforms from the same species. We observed changes at subunit interfaces that were seemingly induced by only six amino acid substitutions. These interface differences likely induced changes in regions of the LSu that are locked into place during substrate (or substrate mimic) binding (Fig. 5*E*). Because cryo-EM captures proteins in their near-native states, we were able to solve our 1A_His_ and 2B/3B_His_ structures in the open state (i.e., without substrate bound) and observe differences in these regions that may have otherwise been masked in a crystal structure (Fig. 5*G*) (43).

Despite high similarity in global structure of our 1A_His_ and 2B/3B_His_ structures (*SI Appendix*, Table S9), we detected key differences at subunit interfaces (Fig. 5 *C* and *D* and *SI Appendix*, Table S10). The two largest interfaces, LL1 and LS1, showed matching trends for area, number of residues, and nonbonded contacts, but an opposite trend for hydrogen bonding. Interface differences resulting from SSus may explain why our measured heterogeneity was less than predicted (Fig. 2*D*), as different SSus binding to opposite ends of an L_2_S_2_ unit (shown in Fig. 5*E*) may reduce its conformational compatibility. However, our recovery of multiple SSu ratios via native PAGE and MS implies that asymmetric L_2_S_2_ units are possible (Fig. 3 *B* and *H*). This is further supported by our companion paper which reports two SSu-heterogeneous structures for *A. thaliana* Rubisco, neither of which follow a 4:4 ratio of SSus (47).

Differences at subunit interfaces may have resulted in the changes in LSu flexibility we observed. Of particular interest is the interaction between the SSu N terminus and LSu loop β8-α8. The SSu N terminus contains a glutamine (1A) to lysine (2B/3B) substitution at residue 2 (*SI Appendix*, Fig. S2), and is ordered in our 2B/3B_His_ structure but flexible in 1A_His_ (Fig. 5*E*). The SSu N terminus contacts LSu loop β8-α8 which shows a similar pattern of differing flexibility between the two structures. LSu loop β8-α8 contributes residues to a phosphate binding site at the active site (48). The loop’s functional importance is supported by the disrupted kinetics observed when mutated, and its flexibility in crystal structures of SSu-lacking Rubisco forms which have a lower affinity for CABP and a greater rate of inhibitor release (37, 49–51).

While the presence of Mg^2+^ in our 1A_His_ structure could have potentially contributed to flexibility and interface differences, structures of active and inactive Rubiscos are highly similar (41, 42). Taylor and Andersson (42) compared structures of activated *S. oleracea* (PDB 1AUS) and unactivated *Nicotiana tabacum* (PDB 3RUB) Rubisco and found no differences in flexibility in regions where we observed large changes (loop β8-α8 and the C terminus of the LSu, and loop βB-αB and the N terminus of the SSu). We extended the analysis of Taylor and Andersson (42) to validate that SSu identity was responsible for interface differences by comparing LL1 interfaces of PDB 1AUS and PDB 3RUB (*SI Appendix*, Table S12) (52). Between the structures, interface area and number of residues increased with Mg^2+^ presence, but number of hydrogen bonds decreased. This is opposite to the trend we observe between our 1A_His_ and 2B/3B_His_ structures with respect to activation, indicating that SSu identity has a larger effect on subunit interfaces than activation status.

Conformational flexibility and activity are often positively correlated, and this relationship has been demonstrated for the Rubisco LSu during the evolution of C_4_ photosynthesis (53, 54). Most Rubiscos from C_4_ plants exhibit higher k_cat,C_ than their C_3_ counterparts (15). The transition from C_3_-like to C_4_-like kinetics requires destabilizing mutations which enhance conformational flexibility in regions that move during catalysis (53). Here, we observe an analogous relationship between flexibility and activity. Although our thermal stability results indicate that SSu1A increases global holoenzyme stability relative to SSu2B/3B, we observe greater flexibility in several residues directly involved in catalysis in our 1A_His_ structure (Fig. 5 *E* and *F*). We posit that SSu1A has a targeted destabilizing effect on Rubisco’s active site relative to SSu2B/3B, enhancing its conformational flexibility to yield an enzyme with higher V_Cmax_ and K_m_^RuBP^, and lower E_a_ for V_Cmax_ (Fig. 4 *B*–*D*). Taken together with our thermal shift data, this suggests active site flexibility is at least partially decoupled from global holoenzyme stability.

Our data indicate that the binding of different SSus to form heterogeneous Rubisco has a linear effect on V_Cmax_ and E_a_ for V_Cmax_, but an antagonistic one on K_m_^RuBP^ and k_obs_ (Fig. 4 *B*–*D* and *F*). As discussed previously, SSu1A and SSu2B/3B potentially caused differences in hydrogen bonding at LL1 and LS1 interfaces. Differing interface conformations could limit the subunit compatibility of a heterogeneous holoenzyme, conferring a destabilizing effect like that we observed in SSu1A-homogeneous Rubisco and causing the higher K_m_^RuBP^ and k_obs_ we measured. However, additional factors may be involved, as we would expect V_Cmax_ and E_a_ for V_Cmax_ to follow a similar pattern if a stability–activity tradeoff were the sole mechanism. Altogether, the significant, albeit subtle structural and kinetic effects of different SSus underscore their importance for the stability and as a result, activity of Form I Rubisco.

### Kinetic Acclimation and the Role of SSu-Heterogeneous Rubisco In Planta.

SSu2B/3B provides a local stabilizing effect relative to SSu1A, as reflected by a lower V_Cmax_ and K_m_^RuBP^, and a higher E_a_ for V_Cmax_ (Fig. 4 *B*–*D*). This aligns with higher relative expression and protein accumulation of the B-family SSus in warmer growth temperatures (21, 22). Warmer temperatures increase the conformational flexibility of proteins (54). For Rubisco, this manifests as higher V_Cmax_, but at the cost of higher K_C_ and lower S_C/O_ (55). The added local stability that B-family SSus provide likely helps limit photorespiration at elevated temperatures, as Rubisco from warm-grown *A. thaliana* has lower K_C_ and higher S_C/O_ relative to that from cold-grown plants (22). This connection between stability and specificity is further supported by the positive correlation between E_a_ for V_Cmax_ and S_C/O_ observed in kinetic surveys (56). For SSu1A, the inverse seems true. By destabilizing the active site, SSu1A may enable greater conformational flexibility at lower temperatures, thereby benefiting the carboxylation capacity of cold-grown plants.

Although we established that SSu-heterogeneous Rubisco exists in planta, its prevalence and role remain unknown. The lower stability suggested by its higher K_m_^RuBP^ may indicate a higher K_C_ and lower S_C/O_, but we did not observe an associated increase in V_Cmax_ that would benefit carboxylation rates. Kinetic surveys show that K_m_^RuBP^ is weakly correlated with other parameters, and thus a more complete kinetic characterization could enable more definite conclusions (15). The higher k_obs_ of SSu-heterogeneous Rubisco could help maintain carboxylation rates at higher temperatures when Rubisco activase is impaired and inhibitors are more abundant (57). However, inhibitor binding plays an important role in regulating Rubisco activity under low light conditions (58). Binding of the naturally occurring inhibitor 2′-carboxy-D-arabitinol 1-phosphate (CA1P) locks Rubisco into a “closed” state, protecting it from proteolytic degradation (59). Therefore, the higher k_obs_ of SSu-heterogeneous Rubisco could make it more susceptible to proteolysis and provide a means to selectively degrade heterogeneous Rubisco.

Our results illustrate a structural mechanism underlying Rubisco kinetic acclimation. As Rubisco often acts as a “bottleneck” in photosynthesis, adapting its kinetics to environmental conditions is paramount to maximizing fitness (23, 56, 60). Here, we follow up on the work of Cavanagh et al. (22) to further characterize the kinetic effect of different SSus in *A. thaliana*. We hypothesize that fine-tuning of local stability underlies the kinetic differences we observed. Most globular proteins are marginally stable at their operating temperature, and deviation from this temperature negatively affects stability and activity (54, 61). A multigene *rbcS* family may enable Rubisco to maintain marginal stability, and therefore activity, over a broader temperature range. Heterogeneity potentially provides an additional mechanism through which stability is manipulated, though its prevalence and role in planta remains elusive.

In addition to temperature, *rbcS* expression varies with other environmental variables such as light and CO_2_, as well as developmental stage and tissue (62–71). Likewise, differences can be observed in Rubisco’s kinetics when assayed from plants grown in different conditions (23, 72). This covariation in expression and kinetics can be observed across a range of plants, indicating SSu-dependent kinetic acclimation may be a mechanism common to many photosynthetic organisms. In summary, our work highlights the kinetic and structural plasticity of Rubisco. Leveraging this plasticity potentially allows for optimization of photosynthesis in a wider range of environmental conditions, and should be explored as an avenue to engineer more resilient and adaptable crops.

## Materials and Methods

For in planta experiments, native *rbcS* ORF sequences were modified by addition of a C-terminal 6xHisTag or StrepTag II, and assembled in tandem with a pFAST-R selection cassette (30) via golden gate cloning (73, 74). Agrobacterium-mediated gene transfer (75) was used to transform *A. thaliana* Col-0, and protein collected from T3 plants used for successive affinity purification. Rubisco expression in *E. coli* relied on the three plasmid system of Ng et al. (28), which is a modified version of that originally described in Aigner et al. (27) and optimized in Wilson et al. (29). Here, the pRSFduet plasmid of the system was altered to add a second *rbcS* with a C-terminal Strep-Tag II for SSu heterogeneity experiments. To quantify heterogeneity, the amount of His- and Strep-tagged L_8_S_8_ Rubisco remaining after each successive affinity purification step was tracked with native PAGE and western blotting. To determine which SSu ratios were present, the pRSFduet plasmid was further altered to add a GB1 tag to the C terminus of one of the SSus, and the resulting purified Rubisco characterized by native and denaturing MS. Activity differences were detected using a spectrophotometric assay (34, 35), with Michaelis–Menten and slow binding inhibition (37, 38) models fit to determine kinetic parameters. Cryo-EM and thermostability analyses were conducted on heterologously expressed 1A_His_ and 2B/3B_His_ Rubiscos. Differences in interfaces and secondary structure between the cryo-EM structures were detected with PDBsum (76) and ChimeraX (77), respectively. Full protocols for cloning, *A. thaliana* growth and transformation, *E. coli* growth, protein purification, PAGE and western blotting, correction for column efficiency, mass spectrometry, kinetics, cryo-EM, and thermal stability analysis are provided in *SI Appendix*.

## Supplementary Material

Appendix 01 (PDF)

Dataset S01 (XLSX)

Dataset S02 (XLSX)

Dataset S03 (XLSX)

Dataset S04 (XLSX)

## Data Availability

Raw mass spectrometry data are available for download at https://doi.org/10.5281/zenodo.15732466 (78). The coordinates of the atomic models of *A. thaliana* Rubisco containing either the 1A or 2B/3B SSu isoforms were deposited in the Protein Data Bank with accession codes 9N37 (39) and 9MUR (40), respectively. All other data are included in the manuscript and/or supporting information.
