# Supplementary material for: Rubisco kinetic acclimation at the holoenzyme level
Source: Proc Natl Acad Sci U S A. 2026 Apr 15;123(16):e2519914123. doi: 10.1073/pnas.2519914123 (PMC13099605; doi:10.1073/pnas.2519914123)
Supplement: Supplementary file 1 — Appendix 01 (PDF) [file pnas.2519914123.sapp.pdf]

PNAS

Supporting Information for
Rubisco kinetic acclimation at the holoenzyme level

Authors:
Bryce Askey[a], Maddie Ceminsky[b], Elena Scott[c], Yongsheng Wang[d], Zhen Guo Oh[a], Stavros Azinas[e], Arthur Laganowsky[c], Laura Helen Gunn[a,b]

Affiliations:
[a]Plant Biology Section, School of Integrated Plant Science, Cornell University, Ithaca, NY, USA.
[b]Biochemistry, Molecular and Cell Biology, Cornell University, Ithaca, NY, USA.
[c]Department of Chemistry, Texas A&M University, College Station, TX, USA.
[d]Department of Molecular Medicine, Cornell University, Ithaca, NY, USA.
[e]Department of Biochemistry and Biophysics, Science for Life Laboratory, Stockholm University, Stockholm, Sweden.

Corresponding authors:
Laura Helen Gunn
lhg42@cornell.edu

This PDF file includes:

SI Methods
Figures S1 to S12
Tables S1 to S12
Legends for Datasets S1 to S4
SI References

Other supporting materials for this manuscript include the following:

Datasets S1 to S4

Table of contents

SI Methods ... **3**
Plasmid design and cloning ... 3
A. thaliana growth, transformation, and protein purification ... 3
E. coli growth, transformation, and protein purification ... 4
PAGE and western blotting ... 5
Correction for column efficiency ... 5
Mass spectrometry ... 6
Kinetics ... 6
Cryo-EM data collection and structural analysis ... 7
Thermal stability analysis ... 8
Supplementary Figures ... **9**
Fig. S1: Affinity purification of SSu-homogeneous controls ... 9
Fig. S2: SSu and LSu sequences with secondary structure annotated ... 10
Fig. S3: *A. thaliana* T1 transformant screening ... 11
Fig. S4: *A. thaliana* T2 transformant screening ... 12
Fig. S5: Western blots of dual SSu purifications ... 13
Fig. S6: Simulated dual purification of affinity tagged Rubisco ... 15
Fig. S7: Mass spectrometry of GB1-free Rubisco ... 17
Fig. S8: Biological replicates of V_C of homo- and heterogeneous Rubisco ... 18
Fig. S9: Cryo-EM data processing workflow for $1A_{His}$ structure ... 19
Fig. S10: Cryo-EM data processing workflow for $2B/3B_{His}$ structure ... 20
Fig. S11: Interface labeling in cryo-EM structures ... 21
Fig. S12: Melting temperature curves from thermal shift assay ... 22
Supplementary Tables ... **23**
Table S1: Primers used for genotyping of *A. thaliana* T2 plants ... 23
Table S2: Native and denatured mass spectrometry instrument parameters ... 24
Table S3: Theoretical and measured masses of homo- and heterogeneous Rubisco ... 25
Table S4: V_{Cmax} and K_m^{RuBP} of homo- and heterogeneous Rubisco ... 26
Table S5: Biological replicates of VC for homo- and heterogeneous Rubisco ... 27
Table S6: E_a for V_{Cmax} of homo- and heterogeneous Rubisco ... 28
Table S7: v_i, v_f, and k_{obs} of homo- and heterogeneous Rubisco ... 29
Table S8: Cryo-EM data collection and refinement parameters ... 30
Table S9: Alignment statistics for superimposed $1A_{His}$ and $2B/3B_{His}$ structures ... 31
Table S10: Interface statistics for $1A_{His}$ and $2B/3B_{His}$ structures ... 32
Table S11: Thermal shift assay-derived thermodynamic values ... 33
Table S12: Interface statistics for PDB 1AUS and PDB 3RUB ... 34
Legends for Supplementary Datasets ... **35**
Dataset S1 (separate file): Metadata for all Form I Rubisco structures uploaded to PDB ... 35
Dataset S2 (separate file): Plasmids used in this study ... 35
Dataset S3 (separate file): Western blotting data used for quantification of His-skew and heterogeneity ... 35
Dataset S4 (separate file): Raw data from kinetic assays ... 35
SI References ... **36**

SI Methods

Plasmid design and cloning

A full list of plasmids is provided in Dataset S2. In accordance with manufacturer directions, BsaI-HF v2 (NEB) and BpiI (Thermo Fisher Scientific) were used for all golden gate reactions, Q5 polymerase (NEB) for all PCR reactions, ClonExpress II Kit (Vazyme) for all infusion reactions, and KLD Enzyme Mix (NEB) for all site-directed mutagenesis (SDM) reactions.

Level 2 plasmids used for transformation of *A. thaliana* plants were assembled in a pAGM4723 backbone via golden gate cloning (1). Golden gate cloning was used to generate the level 2 plasmids used for transformation of *A. thaliana* plants (1). Level 0, 1, and 2 backbones were amplified from the MoClo Toolkit (Addgene), and prior to use, the right border of the level 2 backbone pAGM4723 was corrected by SDM (2). The *rbcS2B* promoter, *rbcS3B* promoter, *NOS* terminator, *OCS* terminator, and pFAST-R selection cassette were amplified from the Plant Parts Kit (Addgene) (3, 4). The *HSP* terminator, and ORF sequences for $rbcS1B_{His}$, $rbcS1B_{Strep}$, $rbcS2B_{His}$, and $rbcS1A_{Strep}$ were ordered as gene fragments from Twist Bioscience. The ORF sequences for all *rbcS* gene fragments were designed to be identical to their native sequences, but with a 6xHis or StrepTag II added to the C-terminus. The *HSP* and *NOS* terminators were used to generate a fused *HSP-NOS* terminator as in Atkinson et al. (5).

Across all *E. coli* expression plasmids, the CDS sequences used for SSu1B and SSu2B/3B were not codon optimized for *E. coli* and are identical to those encoding the mature SSu1B and SSu3B in the *A. thaliana* genome, respectively. However, the SSu1A CDS sequence was codon optimized for *E. coli*.

A pRSFduet-AtRbcLScHis (2B/3B$_{His}$) plasmid (gift from Oliver Mueller-Cajar) was used as a backbone for all cloning (6). To generate dual SSu Rubisco expression plasmids, four gene fragments, representing each SSu combination, were ordered in pET-11a backbones from GenScript. In-Fusion cloning was applied to insert the dual SSu fragments in the pRSFduet-AtRbcLScHis backbone. To generate the expression plasmid for 1A$_{His}$, a gene fragment was ordered from Twist Bioscience and In-Fusion cloning applied to insert into the pRSFduet-AtRbcLScHis backbone. SDM was used to generate the single SSu expression plasmids for 1B$_{His}$ from 1B$_{His}$-1A$_{Strep}$, 1B$_{Strep}$ from 1B$_{Strep}$-1B$_{His}$, 1A$_{Strep}$ from 1A$_{His}$, and 2B/3B$_{Strep}$ from 2B/3B$_{His}$.

To generate the GB1-tagged dual SSu Rubisco expression plasmids, gene fragments were ordered from Twist Bioscience for $rbcS1B_{GB1\text{-}His}$ and $rbcS2B/3B_{GB1\text{-}His}$. In-Fusion cloning was applied to replace the His-tagged SSu with the GB1-His-tagged SSu in the previously generated dual SSu expression plasmids. SDM was used to generate the 2B/3B$_{GB1\text{-}His}$ plasmid from 2B/3B$_{GB1\text{-}His}$-1A$_{Strep}$.

A. thaliana growth, transformation, and protein purification

A. thaliana Col-0 seeds were cold stratified at 4°C for three days on water-soaked filter paper before sowing on potting mix (Miracle-Gro® Potting Mix; ScottsMiracle-Gro). Plants were grown at 22°C, 50% relative humidity, 300 μmol photons m^{-2} s^{-1} in 16:8 h light:dark. To transform plants, level 2 expression plasmids were transformed into *Agrobacterium tumefaciens* GV3101 via electroporation. *A. tumefaciens* was then used for stable transformation of plants via floral dip (7). The pFAST-R selection marker was used to screen for positive transformants in T1 seeds (4). At least four T1 plants per dual-SSu construct were grown to maturity and screened for tagged SSu expression at four weeks via western blotting. Seeds from the T1 line with the strongest western blotting signal were screened for pFAST-R expression using a fluorescent microscope to identify homozygotes. Three four-week-old T2 plants grown from selected seeds were genotyped via PCR to confirm presence and identity of the transgenes (8).

Four sets of primers were used for genotyping (Fig. S2). Set A (primers 17 and 18) amplified a 2639-2660 bp fragment of the transgene from an intron of 2B$_{His}$ to the StrepTag. Set B (primers 19 and 18) amplified a 2282-2303 bp fragment from the HisTag to the StrepTag. Set C (primers 19 and 20) amplified a 2011 bp fragment from the HisTag to an intron of 1A$_{Strep}$. Set D (primers 21 and 22) was designed as a positive control for DNA quality, amplifying a 1650 bp fragment from the actin, *ACT2* gene (AT3G18780). pFAST-R screening of T3 seeds collected from these plants revealed further segregation, indicating silencing and/or multiple transgene insertions in the selected T1 lines. Seeds with the greatest pFAST-R signal were selected and

used to grow plants for dual purification experiments.

Small-scale protein extraction was performed to screen T1 lines for tagged SSu expression. One leaf from each 4-week-old T1 plant was weighed, transferred to a microcentrifuge tube, snap frozen with liquid N_2, and ground with a pestle. Per 1 mg fresh weight, 10 µL plant lysis buffer (20 mM Tris-HCl pH 8.0, 50 mM NaCl, 1 mM EDTA, 5% v/v glycerol, 5 mM DTT, 1% w/v PVPP, 3 % w/v Pierce™ Protease Inhibitor Tablet [Thermo Fisher Scientific], 1 mM PMSF, 2 mM $MgCl_2$, and 25 U/mL Benzonase® Nuclease [Sigma-Aldrich]) was added. After resuspending the ground tissue, soluble and insoluble fractions were separated by centrifugation at 21,100 g, 4°C for 20 minutes. Expression of tagged SSus was measured by western blotting the soluble fraction with HisTag and StrepTag antibodies (Sigma-Aldrich).

A larger-scale extraction was performed to test for the presence of SSu-heterogeneous Rubisco in T3 plants. 6 g of leaves were harvested from four-week-old T3 and WT plants, ground in liquid N_2, and resuspended in 30 mL plant lysis buffer. Lysate was centrifuged at 30,720 g, 4°C for 20 minutes to separate soluble and insoluble fractions. The soluble fraction was filtered through a 0.22 µm syringe filter before being used for purification. This extraction protocol was also used to collect lysate from WT plants for MS analysis, but 18 g of leaves were used instead of 6 g, and the ground tissue was resuspended in 90 mL plant lysis buffer instead of 30 mL.

All protein purifications were performed on an NGC Quest 10 Plus Chromatography System (BioRad). To purify His-tagged Rubisco, filtered lysate was loaded on a HisTrap HP 5 mL column (Cytiva). A sample of soluble lysate was set aside as the "Sol" fraction. The HisTrap was washed with binding buffer (20 mM Tris-HCl pH 8.0, 50 mM NaCl, 1 mM EDTA, 5 mM DTT, 5% v/v glycerol), and then with 90% binding : 10% HisTrap elution buffer (20 mM Tris-HCl pH 8.0, 50 mM NaCl, 1 mM EDTA, 5% v/v glycerol, 5 mM DTT, 500 mM imidazole) until $A_{280\ nm}$ reached a stable baseline. Proteins were eluted with a 10-100% gradient of HisTrap elution buffer over 48 mL, and fractions eluted between 25-85% (32 mL total volume) were collected and pooled. A sample of this pooled eluate was set aside as the "H" fraction.

Pooled HisTrap eluate was then loaded on a StrepTrap HP 5 mL column (Cytiva). The column was washed with binding buffer until $A_{280\ nm}$ reached a stable baseline. Proteins were eluted with 100% StrepTrap elution buffer (20 mM Tris-HCl pH 8.0, 50 mM NaCl, 1 mM EDTA, 5 mM DTT, 5% v/v glycerol, 2.5 mM desthiobiotin), and the first 16 mL of eluate collected. A sample of this pooled eluate was set aside as the "H>S" fraction. Eluate was concentrated and buffer exchanged into storage buffer (20 mM Tris-HCl pH 8.0, 50 mM NaCl, 1 mM EDTA, 5% v/v glycerol) with a 50 kDa MWCO centrifuge filter (Amicon®, Millipore Sigma) For storage, proteins were snap-frozen in liquid N_2 and stored in -80°C.

Anion exchange chromatography was applied to purify untagged Rubisco from WT *A. thaliana* plants for MS analysis. Soluble lysate was loaded on 50 mL (bed volume) of Source 30Q resin packed in a HiScale 26/40 column (Cytiva). The column was washed with 50 mL binding buffer (20 mM Tris-HCl pH 8.0, 50 mM NaCl, 1 mM EDTA, 5% v/v glycerol, 5 mM DTT). Elution buffer (20 mM Tris-HCl pH 8.0, 1 M NaCl, 1 mM EDTA, 5% v/v glycerol, 5 mM DTT) was then applied in a 0-50% gradient over 400 mL. Fractions eluted between 36-40% elution buffer (288-320 mL) were pooled, concentrated, and buffer exchanged into storage buffer before being snap-frozen in liquid N_2 and stored at -80°C.

E. coli growth, transformation, and protein purification

The three plasmid system of Ng et al. (6) was used to express *A. thaliana* Rubisco in *E. coli*. This system is a modified version of that originally described in Aigner et al. (9) and later optimized in Wilson et al. (10). To generate cell lines for Rubisco expression, commercial BL21 (DE3) *E. coli* cells (NEB) were transformed with plasmids p11a-AtC60ab/C20 and pCDFduet-AtR1/R2/Rx/B2, gifts from Manajit Hayer-Hartl. Competent cells were prepared from these cells before subsequent transformation with the appropriate pRSFDuet-1 Rubisco expression plasmid.

All expression media contained 30 µg/mL Kanamycin, 200 µg/mL Ampicillin, and 25 µg/mL Streptomycin for selection. To grow cultures for protein expression, 1 L autoinduction media was inoculated with 20 mL of an overnight culture of BL21 *E. coli* and incubated at 37°C for 6 h (11). The culture was then transferred to 23°C and incubated for 18 h. Cells were harvested via centrifugation at 8,000 x g, 4 °C for 30 min. 1 L pellets were resuspended in 20 mL lysis buffer (20 mM sodium phosphate pH 8.0, 500 mM NaCl, 3 % w/v Pierce™ Protease Inhibitor

Tablet [Thermo Scientific], 1 mM PMSF, 2 mM $MgCl_2$, and 25 U/mL Benzonase® Nuclease [Sigma-Aldrich]). Resuspended cells were lysed by French Press (GlenMills) at 6895 kPa, and soluble and insoluble fractions separated via centrifugation at 30,720 x g, 4°C for 20 min. The soluble fraction was filtered through a 0.22 µm syringe filter before being used for purification.

The same His- and StrepTrap protocols used to purify SSu-heterogeneous Rubisco from *A. thaliana* plants were applied to purify *E. coli*-expressed Rubisco. However, different binding buffer (20 mM sodium phosphate pH 8.0, 500 mM NaCl), HisTrap elution buffer (20 mM sodium phosphate pH 8.0, 500 mM NaCl, 500 mM imidazole), and StrepTrap elution buffer (20 mM sodium phosphate pH 8.0, 500 mM NaCl, 2.5 mM desthiobiotin) were used. Additionally, for purifications used to quantify heterogeneity, soluble protein concentrations were first measured with a Pierce™ Bradford Protein Assay Kit (Thermo Fisher Scientific), and used to prepare 16 mL soluble lysate at 7.5 mg/mL (120 mg total protein). A sample of this diluted soluble lysate was set aside as the “Sol” fraction.

For Rubisco undergoing only HisTrap purification ($1B_{His}$, $2B/3B_{His}$, and $2B/3B_{GB1\text{-}His}$), pooled HisTrap eluate was concentrated and buffer exchanged into storage buffer as described for purified protein from *A. thaliana* plants. For Rubisco undergoing only StrepTrap purification ($1B_{Strep}$ and $1A_{Strep}$), lysate was instead loaded directly onto the StrepTrap, and pooled StrepTrap eluate concentrated and buffer exchanged.

$1B_{His}$ and $2B/3B_{His}$ were used to verify specificity of the StrepTrap for Strep-tagged protein, and $1B_{Strep}$ and $1A_{Strep}$ to verify specificity of the HisTrap for His-tagged protein. Lysate representing these Rubiscos was subjected to the same dual purification protocol as SSu-heterogeneous Rubisco. However, the order of purification was swapped for $1B_{Strep}$ and $1A_{Strep}$. Therefore, the purification samples collected are designated “Sol”, “S”, and “S>H”.

The same HisTrap protocol was also applied to purify $2B/3B_{His}$ Rubisco for cryo-EM. However, expression and purification of $1A_{His}$ Rubisco for cryo-EM differed slightly. For this Rubisco, a 2 L culture was grown in autoinduction media and after harvesting, cells were lysed by sonication. The HisTrap purification method was adjusted so that the 10-100% gradient elution step spanned 30 mL instead of 48 mL. Fractions from 22%-49% HisTrap elution buffer (4-13 mL) during gradient elution were collected and pooled for further purification via size exclusion. 8 mL of the pooled HisTrap fractions were loaded on a HiLoad 26/600 Superdex 200 pg column (Cytiva) and eluted with binding buffer. Fractions from 90-160 mL were pooled, concentrated, and buffer exchanged into HEPES buffer (100 mM HEPES pH 8.5, 10 mM $MgCl_2$, 1 mM EDTA, 10 mM $NaHCO_3$) before being analyzed by cryo-EM.

PAGE and western blotting

Native and SDS PAGE were used to assess Rubisco purity during purification. Native PAGE used 7.5% Mini-PROTEAN TGX precast polyacrylamide gels (Bio-Rad), or 6% 0.75 mm hand-cast polyacrylamide gels. SDS PAGE used 8-16% Mini-PROTEAN TGX precast polyacrylamide gels (Bio-Rad). Gels were stained with G-250 stain (40% v/v ethanol, 8% v/v acetic acid, 0.1% w/v Coomassie Brilliant Blue G-250 [Bio-Rad]), and destained with destaining buffer (10% v/v ethanol, 7% v/v acetic acid).

Western blotting was used to screen T1 *A. thaliana* lines for expression of affinity-tagged SSus and to quantify the heterogeneity of heterologously expressed Rubiscos. Following separation on SDS or native PAGE, proteins were wet-transferred to a nitrocellulose membrane in a Mini Trans-Blot Cell (Bio-Rad). Membranes were blocked with 5% w/v nonfat milk powder dissolved in TBS-T (20 mM Tris base pH 8.0, 150 mM NaCl, 0.05% v/v Tween-20) for 1 h. After blocking, monoclonal antibodies specific to either the 6xHisTag or StrepTag II (Sigma-Aldrich) were prepared at a 1:3000 dilution in TBS-T and applied to the membranes. After incubating overnight at 4°C, membranes were rinsed thrice for 10 min with TBS-T and incubated at 23°C with a monoclonal horseradish peroxidase-conjugated secondary antibody (Thermo Fisher Scientific) at a 1:10000 dilution in TBS-T. Membranes were rinsed thrice for 10 min with TBS-T, and Pierce ECL Western Blotting Substrate (Thermo Fisher Scientific) applied for visualization. All gels and immunoblots were imaged with a ChemiDoc Imaging System (Bio-Rad).

Correction for column efficiency

Rubisco concentrations following each purification step were measured via western

blotting, and used to quantify heterogeneity of dual SSu Rubiscos over 6 biological replicates. To do this, Sol, H, and H>S fractions were ran on native PAGE alongside a dilution series of 1, 0.5, 0.2, 0.1, 0.05, and 0.02 µg of purified SSu-homogeneous Rubisco. To ensure all loaded samples were within the range of the dilution series, the Sol sample was diluted 1:8, the H sample 1:4, and the H>S sample kept at its original concentration. Following separation on native PAGE, proteins were transferred to a nitrocellulose membrane, antibodies applied and visualized, and the membrane imaged (full protocol in SI Appendix). ImageJ was used to quantify band intensities, and after accounting for loading volume and dilution, uncorrected protein amounts calculated using a linear model fit to the dilution series (12).

A correction for column efficiency was required to calculate the true amount of His- and Strep-tagged Rubisco in total soluble protein (Sol_{His} and Sol_{Strep}), after HisTrap purification (H_{His} and H_{Strep}), and after His- and StrepTrap purification ($H>S_{His}$ and $H>S_{Strep}$). Efficiency was quantified as the fraction of appropriately tagged Rubisco surviving each purification step. For example, HisTrap efficiency is given by $H_{His,uncorrected}$ / Sol_{His}. Corrected amounts for His- and Strep-tagged Rubisco are then calculated as $H_{His} = H_{His,uncorrected} / (H_{His,uncorrected} / Sol_{His})$ and $H_{Strep} = H_{Strep,uncorrected} / (H_{His,uncorrected} / Sol_{His})$. Likewise, StrepTrap efficiency is given by $H>S_{Strep,uncorrected} / H_{Strep}$, and corrected amounts calculated as $H>S_{Strep} = H>S_{Strep,uncorrected} / (H>S_{Strep,uncorrected} / H_{Strep})$ and $H>S_{His} = H>S_{His,uncorrected} / (H>S_{Strep,uncorrected} / H_{Strep})$. To account for variability in HisTrap and StrepTrap efficiency across purifications, column efficiency was calculated and corrected per-purification (i.e., per-biological replicate). With corrected protein amounts, His-skew was calculated as Sol_{His} / Sol_{Total}, where $Sol_{Total} = Sol_{His} + Sol_{Strep}$, and heterogeneity as $H>S_{Total} / Sol_{Total}$. A one-sample t-test with significance cutoff 0.05 was used to test for constructs with His-skew significantly different from 0.5. One-way ANOVA and post-hoc Tukey HSD with cutoff 0.05 were applied to detect significant differences in heterogeneity.

The accuracy of our column efficiency correction was evaluated using a simulated dataset with varying degrees of His-skew in R (13). This dataset was generated by assuming the Rubisco population in each sample follows a binomial distribution with n = 8 and p = His-skew, with an additional amount added to the SSu-homogeneous pools at the extremes of the distribution. Sequential HisTrap and StrepTrap purification was simulated with varying base purification efficiency (Offset) and dependence on the number of appropriate tags per holoenzyme (Regularity) (Fig. S6).

Mass spectrometry

Proteins for native MS study were prepared as described in Laganowsky et al. (14). In brief, proteins were buffer exchanged into 200 mM ammonium acetate with a centrifugal desalting column (Micro Bio-Spin 6, Bio-Rad). Denatured samples composed of a final amount of 25% acetonitrile. Samples were then loaded into pulled borosilicate glass capillaries and electrosprayed with a voltage applied through a platinum wire inserted directly into the solution inside the glass capillary. Ionized samples were then introduced into Q Exactive UHMR Hybrid Quadrupole-Orbitrap Mass Spectrometer (Thermo Fisher Scientific). Tuning parameters for data collection are listed in Table S2. Native MS data were deconvoluted and analyzed using UniDec (15).

Kinetics

A modified GAPDH-GlyPDH spectrophotometric assay was used to measure Rubisco carboxylation rate (16, 17). In this modified version, the reaction chain is truncated after the formation of glyceraldehyde-3-phosphate, meaning two molecules of NADH are oxidized per RuBP consumed. To prepare activated Rubisco (ECM) for the assay, an NP80 spectrophotometer (Implen) was first used to measure the concentration of purified Rubisco, with the non-reduced extinction coefficient used to calculate active site concentration from $A_{280\ nm}$. ECM was prepared by incubating 5 µM Rubisco active sites in 20 mM Tris-HCl pH 8.0, 50 mM NaCl, 10 mM $MgCl_2$, and 40 mM $NaHCO_3$ for 1 h at 23°C. Prior to taking measurements, ECM, assay buffer (final assay concentration 100 mM Tricine-KOH pH 8.0, 0.5 mM NADH, 1 mM ATP, 10 mM phosphocreatine, 20 mM $NaHCO_3$, 10 mM $MgCl_2$, 25 U/mL 3-phosphoglyceric phosphokinase [Sigma-Aldrich], 25 U/mL creatine kinase [Sigma-Aldrich], 25 U/mL glyceraldehyde-3-phosphate dehydrogenase [Sigma-Aldrich]), and water were placed on a heat block set to 25°C or 35°C. 65 µL assay buffer was added to a quartz Ultra-Micro Cell cuvette

(Hellma) preheated to 25°C or 35°C, and varying volumes of a 32 mM, 5 mM, or 1 mM RuBP stock solution (gift from Oliver Mueller-Cajar) added to bring the final assay concentration to 1, 0.5, 0.2, 0.1, 0.05, or 0.02 mM RuBP. Water was used to bring the volume to 90 µL, and 10 µL ECM (final assay concentration 0.5 µM active sites) mixed in to initiate the reaction. $A_{340\ nm}$ was tracked over 120 s with a Cary 60 UV-Vis spectrophotometer (Agilent) with water bath set to 25°C or 35°C.

Model fitting and statistical analyses were performed in R (13). A linear model was fit to the linear portion of $A_{340\ nm}$ for each reaction. For reactions with 1 or 0.5 mM RuBP, this was the first 60 s. For reactions with 0.2 or 0.1 RuBP, the first 40 s. And for reactions with 0.05 or 0.02 RuBP, the first 20 s. RuBP consumption in µmol s^{-1} was calculated from the slope of each linear model using equation 1 from Sales et al. (18), and divided by the number of active sites to give carboxylation rate in s^{-1}. A Michaelis-Menten model was fit to each technical replicate (the same purified Rubisco measured 6 RuBP concentrations) to calculate V_{Cmax} and K_m^{RuBP}.

A similar spectrophotometric assay was used to measure the rate of inhibitory RuBP release. RuBP-inhibited Rubisco (ER) was prepared by incubating 5 µM Rubisco active sites with 2.5 mM EDTA for 10 minutes at 23°C. RuBP was added to 1 mM and the mixture incubated at 23°C for an additional 50 minutes. The spectrophotometric assay was then performed at 25°C as described previously, with RuBP added to a final assay concentration of 1 mM. $A_{340\ nm}$ was tracked over 600 s for 5 technical replicates and used to calculate cumulative RuBP consumption (equivalent to CO_2 fixed) per active site over the course of the assay. Equation 1 from Pearce and Andrews (19) was fit to cumulative RuBP consumption for each replicate and used to calculate initial and final carboxylation rates (v_i and v_f, respectively), and the first order rate constant for inhibitory RuBP release (k_{obs}). To aid model convergence, v_i, v_f, and k_{obs} were initialized at 0 s^{-1}, 0.25 s^{-1}, and 0.001 s^{-1}, respectively. One-way ANOVA and post-hoc Tukey HSD with cutoff 0.05 were applied to detect significant differences in kinetic parameters.

Cryo-EM data collection and structural analysis

For cryo-EM analysis of $1A_{His}$ Rubisco, purified protein was incubated with 10 mM $MgCl_2$ and 10 mM $NaHCO_3$ for 30 minutes at room temperature. Quantifoil Cu R2/2 300 mesh grids were glow discharged for 30 seconds. Using a Vitrobot Mark IV (FEI), grids were blotted for 3 seconds with 3 µL of 1 mg/mL sample (100% humidity, 4 °C) and plunge frozen in liquid ethane. Data was collected on a Titan Krios G3i (Thermo Fisher Scientific) microscope using a K3 detector (Gatan) in super-resolution hard-binned mode. 2976 movies were obtained in super-resolution at 130kX, corresponding to a pixel size of 0.664 Å. Movies consist of 40 frames with total dose 40 e-/Å^2 and were imaged at a defocus range between −0.6 and −2.4 µm.

Movies were imported into Cryosparc v3.0 (20), where motion correction and dose weighting were performed and contrast transfer function (CTF) was estimated. Particles were picked from 500 micrographs using Blob Picker, then extracted for 2D Classification. 2D classes were used as templates to pick particles for the whole dataset. Particles were extracted and binned to a pixel size of 6 Å. 2D Classification was performed and high-quality classes were used for Ab Initio volume generation. The resulting volumes were subjected to iterative heterogeneous and homogeneous refinements in C1. The final set of particles were unbinned and refined using Non-Uniform Refinement with D4 symmetry imposed (21). Resolution was estimated via tight mask gold-standard FSC 0.143 cutoff in Cryosparc. The volume was initially fit into PDB 5IU0 using UCSF ChimeraX (22), then further refined using Coot (23). A final real space refinement was performed using Phenix (24).

For cryo-EM analysis of $2B/3B_{His}$ Rubisco, 400-mesh Quantifoil 1.2/1.3 gold grids with 2 nm carbon support (Electron Microscopy Sciences) were glow discharged for 30 seconds. Using a Vitrobot Mark IV (FEI), grids were blotted for 4 seconds with 4 µL of 0.5 mg/mL purified protein (100% humidity, 4 °C) and plunge frozen in liquid ethane. Grids of appropriate quality were imaged using a Talos Arctica 200 kV (Thermo Fisher Scientific) microscope equipped with a K3 detector (Gatan) and the EPU software (Thermo Fisher Scientific). 1229 movies were obtained at 79kX, corresponding to a pixel size of 1.07 Å. Movies consist of 40 frames with total dose 50 e-/Å^2 and were imaged at a defocus range between −0.5 and −2.5 µm. The spherical aberration of the objective lens was 2.7 mm with an objective aperture of 100 µm.

Movies were imported into Cryosparc v4 (20, 25). Motion correction was performed with Patch Motion Correction and CTF was estimated with Patch CTF Estimation. Particles from two micrographs were manually picked, extracted, and subjected to 2D classification, then used as templates to pick particles from all micrographs. Picked particles were extracted and subjected to iterative 2D classification. Final classes were used for Ab Initio volume generation. The resulting volumes were subjected to iterative heterogenous refinement, homogeneous refinement, and 2D classification in C1 and D4. To resolve the active site, symmetry expansion and local refinement were performed with a local mask over the active site. The volume was subjected to 3D classification with 10 classes, three of which were pooled. Duplicate particles were removed, and a final homogenous refinement was performed in D4 symmetry. Resolution was estimated via corrected gold-standard FSC 0.143 cutoff in Cryosparc and local resolution was estimated in Cryosparc (20). The volume was fit into the 1A model using UCSF ChimeraX (22) and further fit and refined using Coot (23). A final refinement was performed using Phenix (24).

Structures were superimposed using Gesamt in the CCP4 suite (26, 27). Additional analysis was performed via PDBsum (28). Visualization performed with UCSF ChimeraX (22).

Thermal stability analysis

Method performed as in Joiner and Fromme (29). Rubisco was diluted to 5 µM final concentration (active site basis) in TSA buffer (50 mM NaCl, 20 mM Tris-HCl, pH= 8.0) containing SYPRO Orange (Thermo Fisher Scientific; 1:1667 dilution of a 5000X concentrate). For measurements of the active and inhibited states, samples were preincubated for 50 minutes with either 10 mM $MgCl_2$ and 10 mM $NaHCO_3$ (active state) or 4 mM EDTA and 1 mM RuBP (inhibited state). Fluorescence signals were measured using a Roche 480 LightCycler while gradually increasing the temperature. Each fluorescence trace represents the average of three replicates, with shaded regions indicating the standard deviation. The relative fluorescence signal (P) was normalized to a range of 0 to 1. Gibbs free energy of unfolding $\Delta G_{unfolding}$ at each temperature was calculated using the equation:

$$\Delta G_{unfolding} = -RTln(\frac{P}{1-P});$$

Furthermore, Gibbs free energy was expressed as:

$$\Delta G_{unfolding} = \Delta H_{unfolding} - T\Delta S_{unfolding}$$

A linear regression model was applied to determine $\Delta H^0_{unfolding}$ and $\Delta S^0_{unfolding}$, allowing calculation of $\Delta G^0_{unfolding}$. The melting temperature $T_m = \frac{\Delta H^0_{unfolding}}{\Delta S^0_{unfolding}}$. One-way ANOVA and post-hoc Tukey HSD with cutoff 0.05 were applied to detect significant differences.

Supplementary Figures

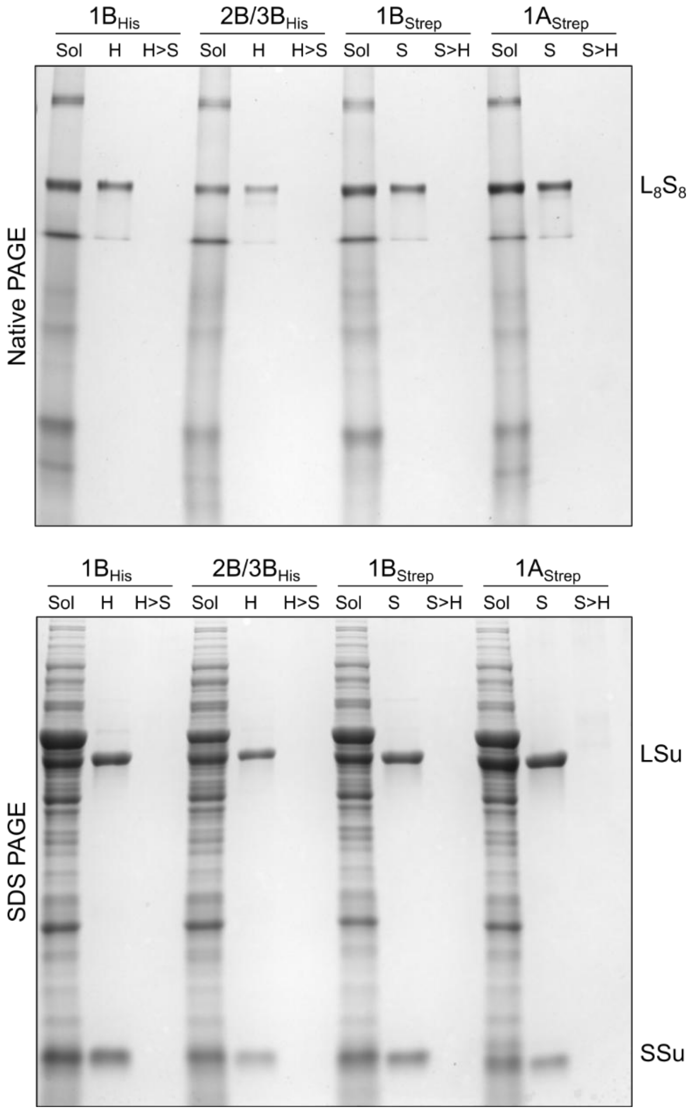

Fig. S1: Affinity purification of SSu-homogeneous controls
Native and SDS PAGE of total soluble and affinity-purified lysate from single SSu-expressing *E. coli*. No protein remains after dual purification, validating column specificity. Sol: total soluble; H: HisTrap purified; H>S: HisTrap and StrepTrap purified; S: StrepTrap purified; S>H StrepTrap and HisTrap purified.

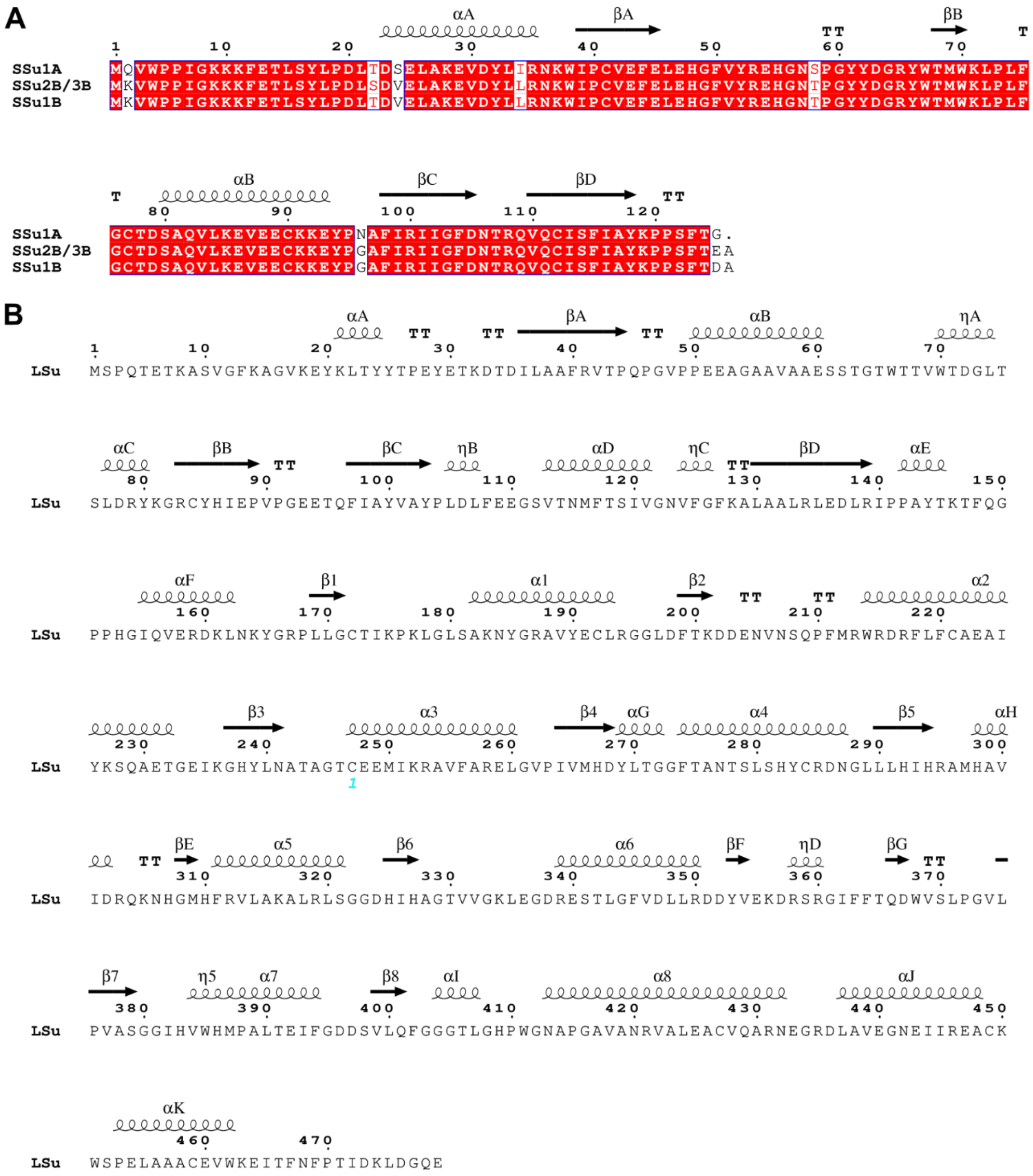

Fig. S2: SSu and LSu sequences with secondary structure annotated
(A) Sequence-structure alignment of mature SSu1A, SSu2B/3B, and SSu1B from *A. thaliana*. Conserved residues are boxed, well-conserved residues are shown in red text, and strictly conserved residues are shown with a red background and white text. Dots indicate gaps. PDB entry 5IU0 was used to annotate secondary structure: α, α-helix; β, β-strand; TT, strict β-turn. The alignment was generated with T-Coffee Expresso using UniprotKB accession numbers P10795 (SSu1A), P10797 (SSu2B/3B), and P10796 (SSu1B) (30). (B) LSu sequence with structural annotations from PDB entry 5IU0. ESPript 3.0 used to visualize sequences (31).

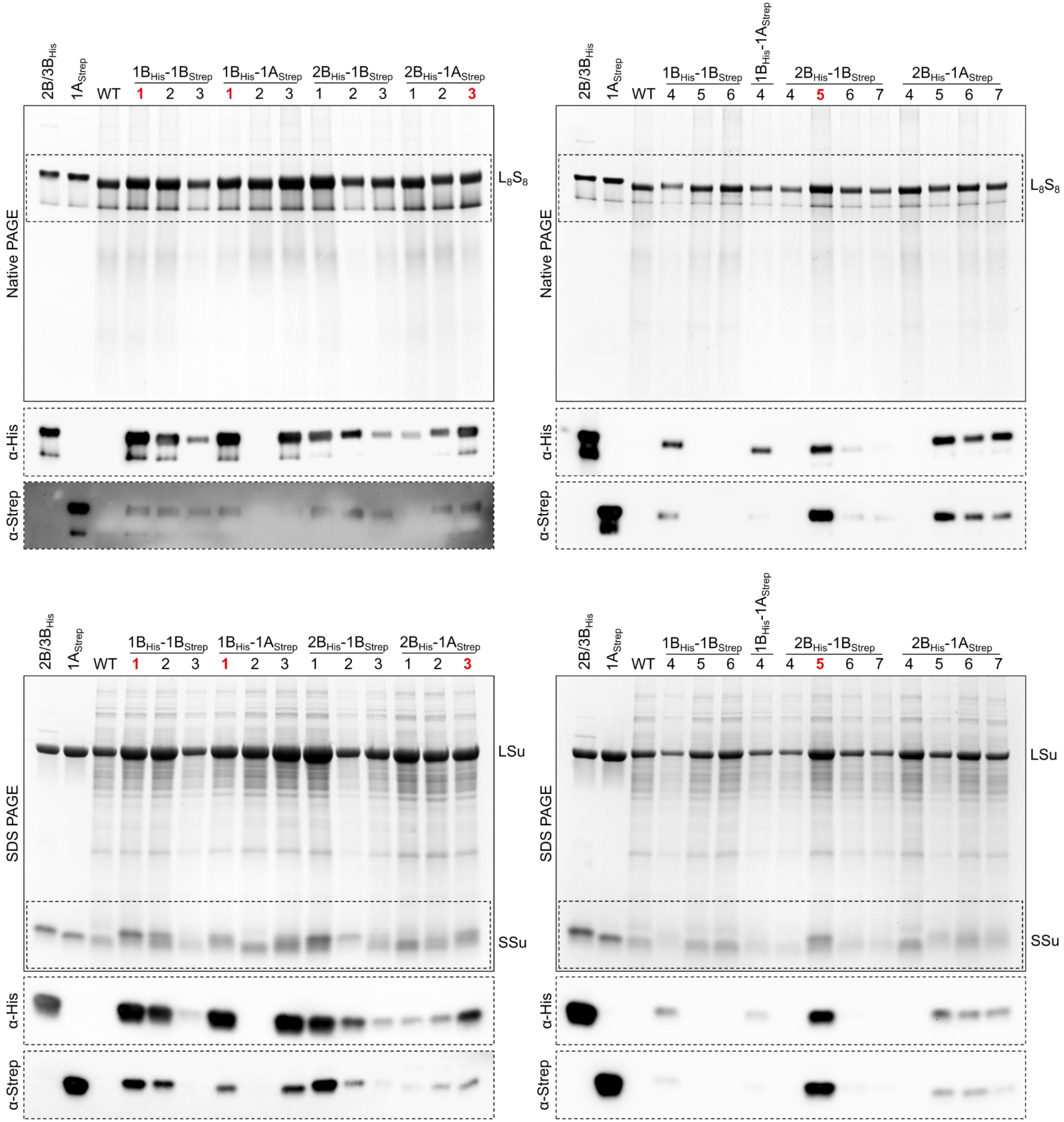

Fig. S3: *A. thaliana* T1 transformant screening
Soluble protein was collected from leaf tissue of four to seven lines per dual SSu construct. Native and SDS PAGE followed by western blotting were then used to screen *A. thaliana* T1 lines for expression of His- and Strep-tagged SSus. Red, bold text indicates the line carried forward through T2 and T3 generations. Heterologously expressed and purified 2B/3B$_{His}$ and 1A$_{Strep}$ Rubisco were loaded as controls.

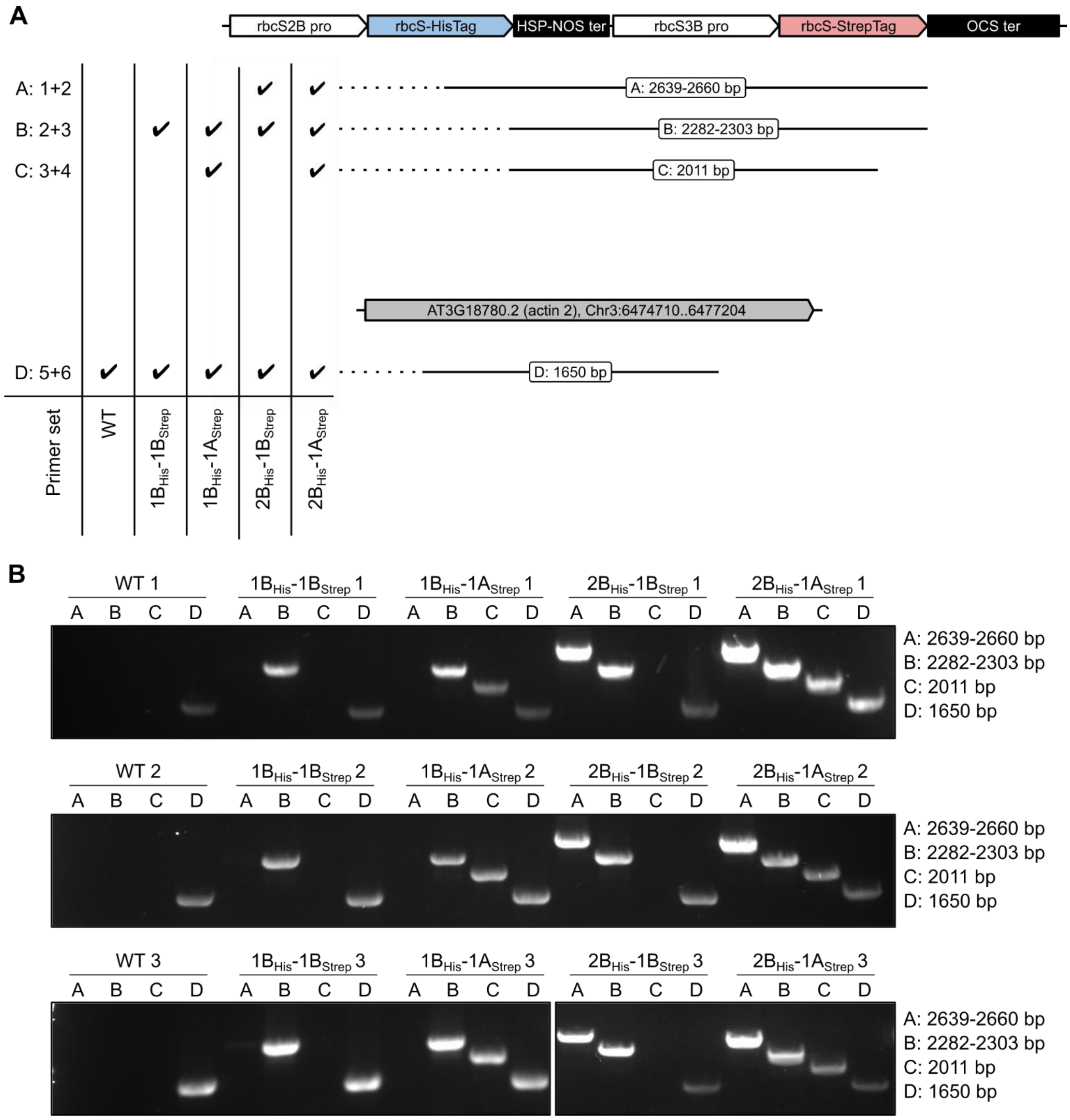

Fig. S4: *A. thaliana* T2 transformant screening
(A) Primer binding locations in transgene insert used for genotyping. Three sets of primers uniquely identify each construct. Depending on the construct, primer set A amplifies a 2639-2660 bp fragment, primer set B a 2282-2303 bp fragment, primer set C a 2011 bp fragment, and primer set D a 1650 bp fragment. Primer set D amplifies a portion of the actin 2 gene as a positive control.
(B) DNA gels from three individual *A. thaliana* T2 plants per construct from which seeds were collected to grow the T3 generation. Lane labels (A-D) indicate the primer set used.

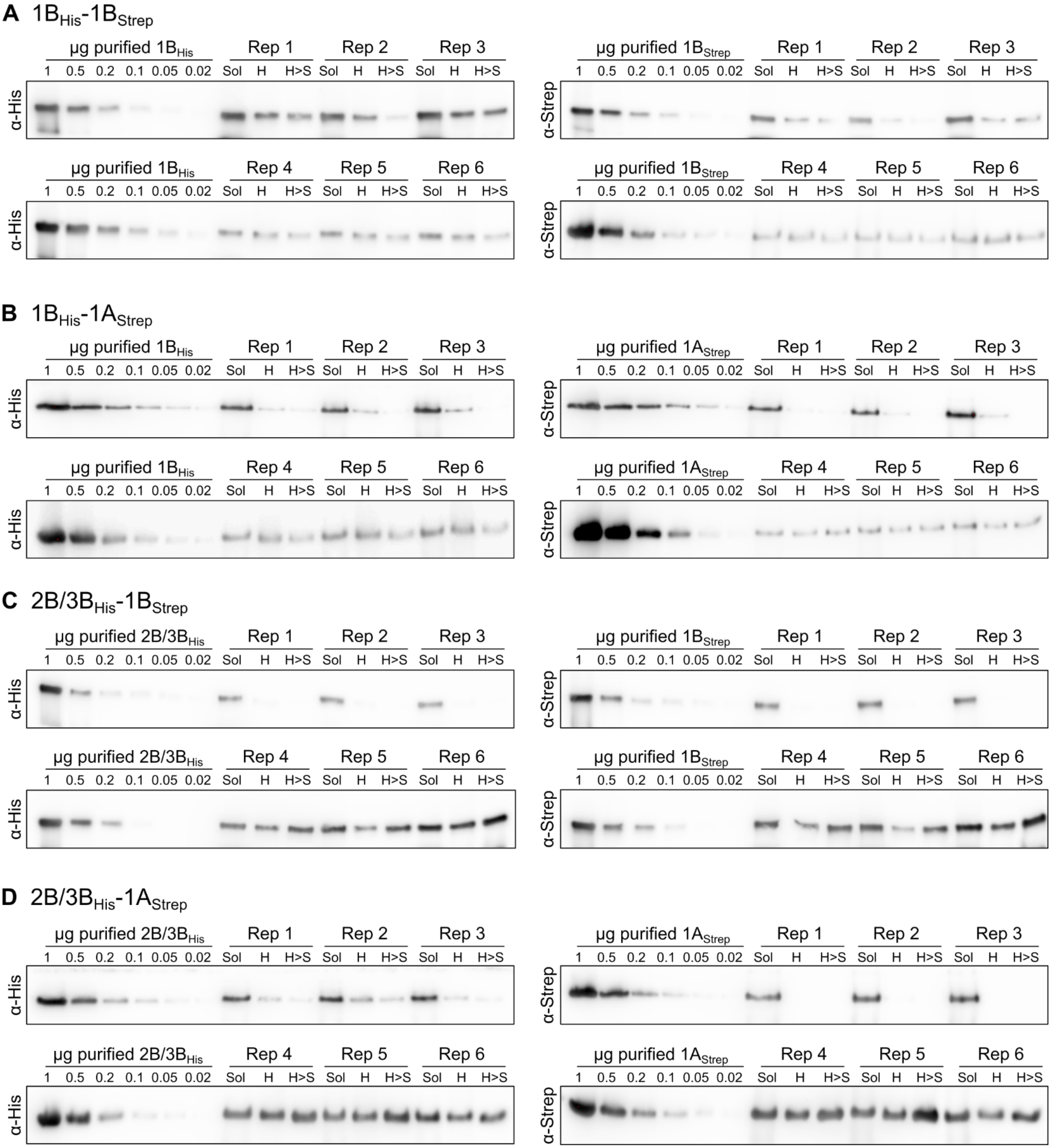

Fig. S5: Western blots of dual SSu purifications

Native PAGE western blots probed with His- and StrepTag antibodies track the amount of Rubisco remaining after each purification step for six biological replicates of (A) $1B_{Strep}$-$1B_{His}$, (B) $1B_{His}$-$1A_{Strep}$, (C) $2B/3B_{His}$-$1B_{Strep}$, and (D) $2B/3B_{His}$-$1A_{Strep}$ Rubisco. Purified SSu-homogeneous Rubisco was loaded as a dilution series and used to convert band intensities to protein concentrations. More protein appears to be retained after HisTrap purification in replicates 4-6 because these were run on a fresh HisTrap column.

However, differences in column efficiency are accounted for in our correction method used to calculate heterogeneity. Sol: total soluble; H: HisTrap purified; H>S: HisTrap and StrepTrap purified.

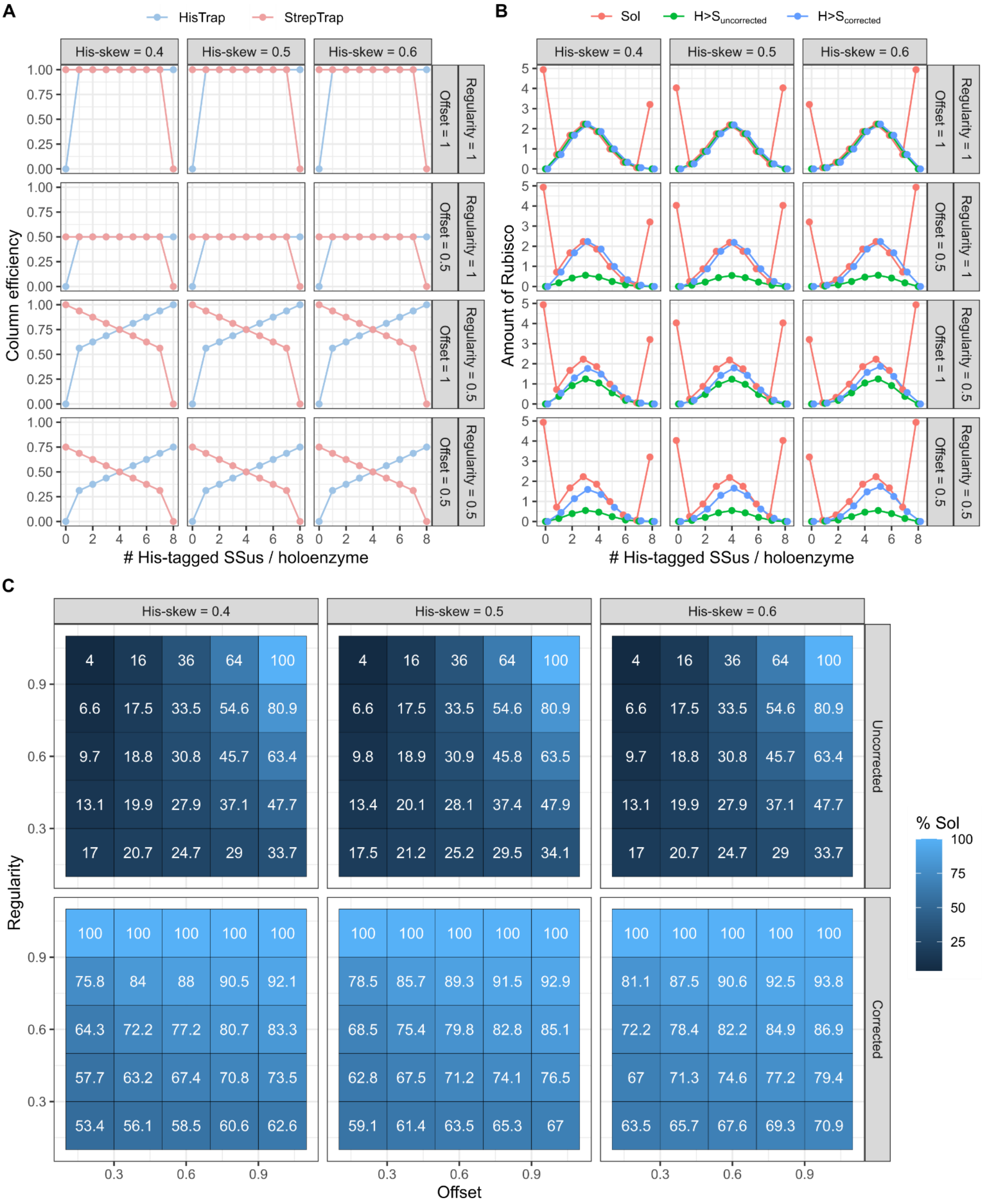

Fig. S6: Simulated dual purification of affinity tagged Rubisco
Testing our correction method on a simulated dataset demonstrates that it accurately recovers the initial amount of SSu-heterogeneous Rubisco when column efficiency is constant (Regularity = 1). (A) Simulated column efficiency with varying Regularity and overall efficiency (Offset). Regularity = 0.5 indicates that column efficiency drops by 50% as the number of appropriately tagged SSus decreases from eight to zero. Each row represents a purification scenario (parameterized by Regularity and Offset), and each column an expression scenario (parameterized by His-skew). Testing these three parameters enabled identification of those which affect the accuracy of our correction method. (B) Simulated input population of Rubisco (Sol) with varying levels of His-skew, and predicted amounts without ($H{>}S_{uncorrected}$) and with ($H{>}S_{corrected}$) correction. (C) Accuracy of heterogeneity quantification without and with correction applied, simulated for varying His-skew, Regularity, and Offset. Accuracy is quantified by % Sol, which is calculated

as the percentage of the predicted amount of SSu-heterogeneous Rubisco in the SoI sample by the true amount. If purification Regularity is less than 1, our correction method underestimates the true amount of SSu-heterogeneous Rubisco, and this underestimation is exacerbated by lower Offset and His-skew.

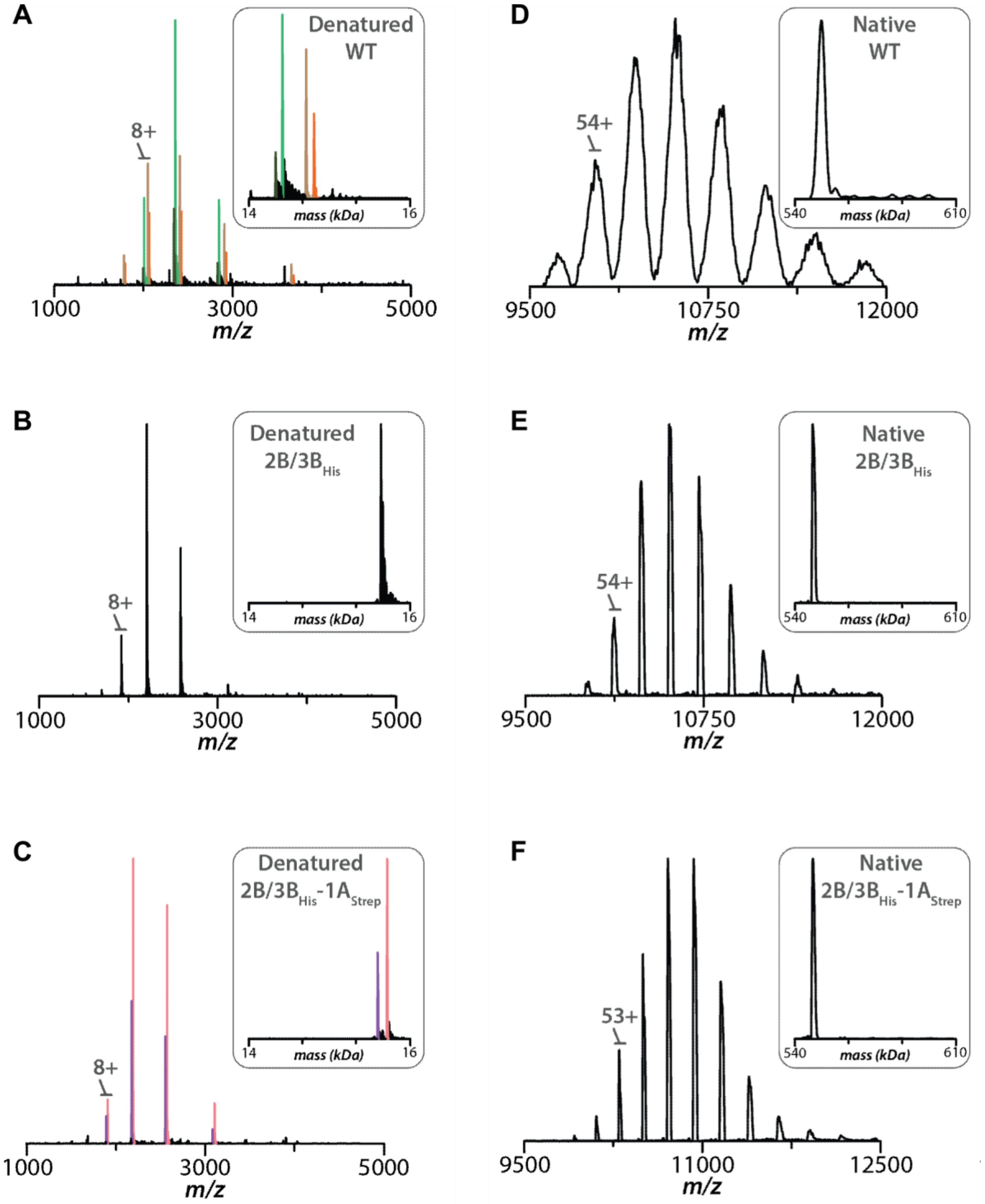

Fig. S7: Mass spectrometry of GB1-free Rubisco
Without an added GB1 tag, mass differences between Rubiscos with different SSu ratios are too small for our MS method to resolve. MS spectra of denatured (A) *A. thaliana* Rubisco from WT plants, (B) $2B/3B_{His}$ Rubisco from *E. coli*, and (C) $2B/3B_{His}$-$1A_{Strep}$ Rubisco from *E. coli*. MS spectra of native (D) *A. thaliana*, (E) $2B/3B_{His}$, and (F) $2B/3B_{His}$-$1A_{Strep}$ Rubisco. Deconvoluted spectra in boxed window of each plot. *A. thaliana* and $2B/3B_{His}$-$1A_{Strep}$ samples contain multiple SSus as illustrated in denatured spectra, but only 1 holoenzyme population can be resolved in their respective native spectra.

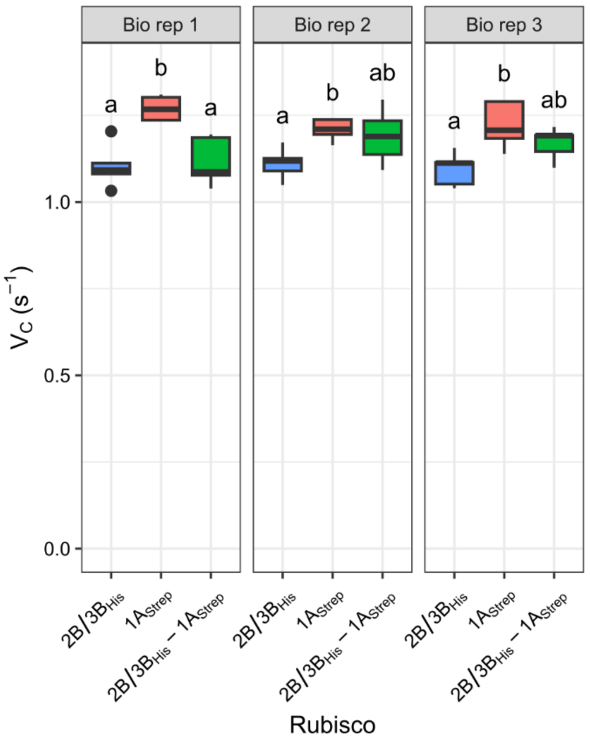

Fig. S8: Biological replicates of V_C of homo- and heterogeneous Rubisco
Spectrophotometric assay-measured V_C at 25°C with 1000 μM RuBP for SSu-heterogeneous 2B/3B$_{His}$-1A$_{Strep}$ Rubisco and its pure counterparts. Each panel contains data from a different biological replicate, representing a separate Rubisco purification and preparation of the assay enzyme mixture. 5 technical replicates were collected per biological replicate. Letters indicate ranking of means following one-way ANOVA and post-hoc Tukey HSD test with significance cutoff 0.05. A separate ANOVA and post-hoc Tukey HSD test were conducted per biological replicate.

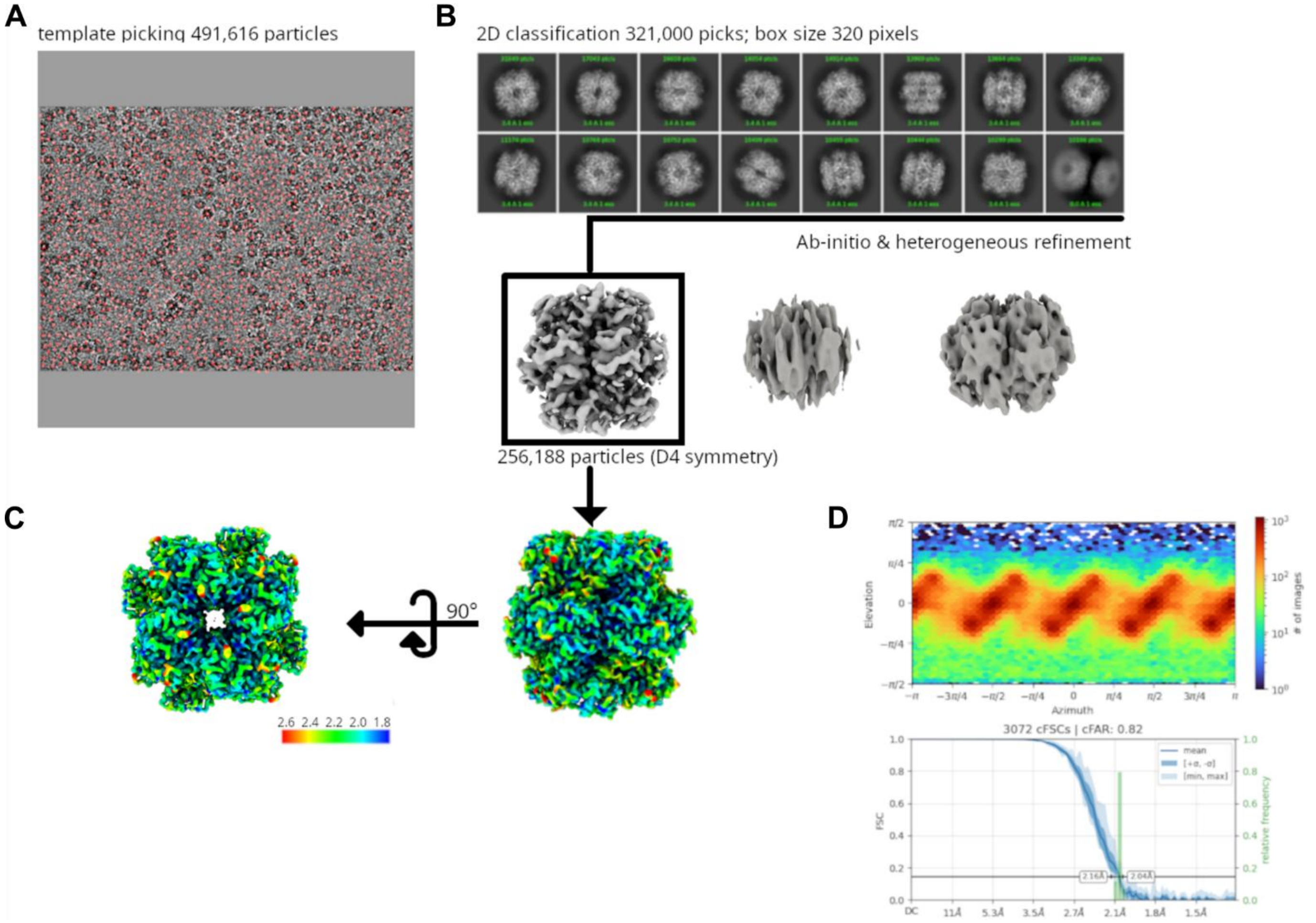

Fig. S9: Cryo-EM data processing workflow for 1A$_{His}$ structure
(A) Representative micrograph showing particle picks for 1A$_{His}$ structure. (B) Workflow for processing structure. (C) Local resolution map. (D) Orientation distribution and cFSC curve for final model. PBD: 9N37.

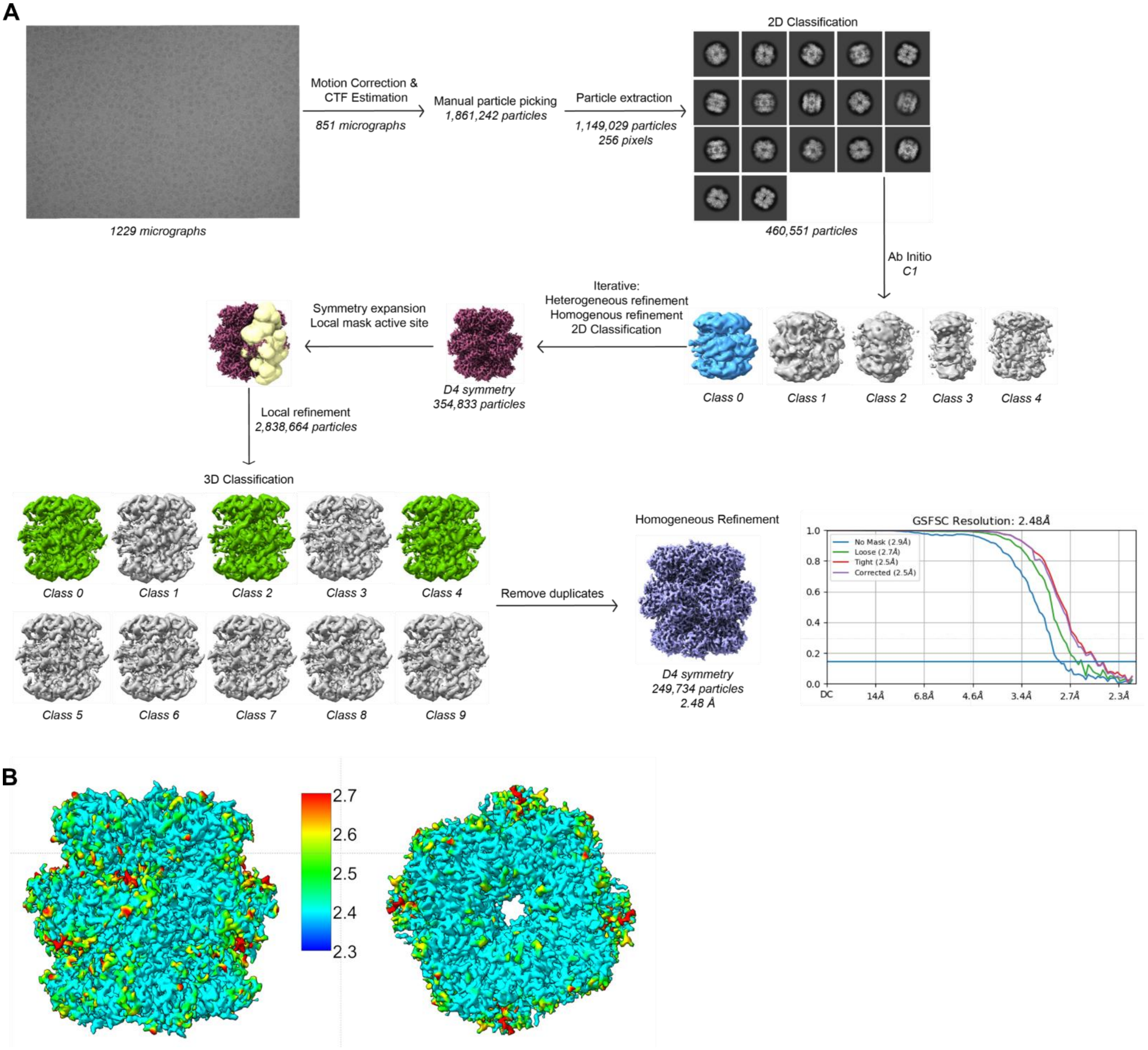

Fig. S10: Cryo-EM data processing workflow for 2B/3B$_{His}$ structure
(A) Workflow and FSC curve for processing 2B/3B$_{His}$ structure. (B) Local resolution map for 2B/3B$_{His}$ structure. PDB: 9MUR.

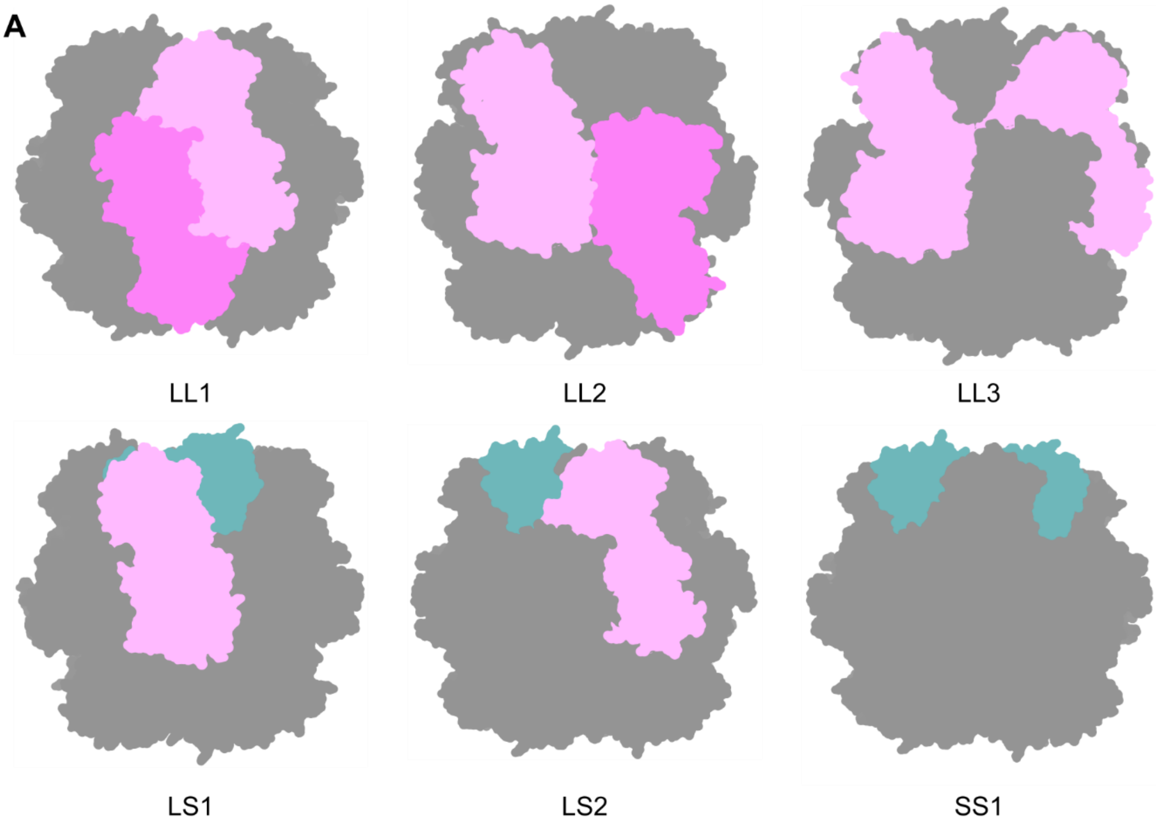

B

Interface	Chains
LL1	AB, CD, EF, GH
LL2	AH, BC, DE, FG
LL3	AC, BD, CE, DF, EG, FH, GA, HB
LS1	AI, BJ, CK, DL, EM, FN, GO, HP
LS2	AK, BP, CM, DJ, EO, FL, GI, HN
SS1	IK, JP, KM, MO, IO, JL, LN, NP

Fig. S11: Interface labeling in cryo-EM structures
(A) Cartoons of subunit interfaces in Rubisco. Terminology from van Lun et al. (2014). L indicates LSu, which are shown in pink. S indicates SSu, which are shown in teal. (B) Interface-forming chains in $1A_{His}$ and $2B/3B_{His}$ Rubisco structures. Chains A-H are LSus and I-P are SSus.

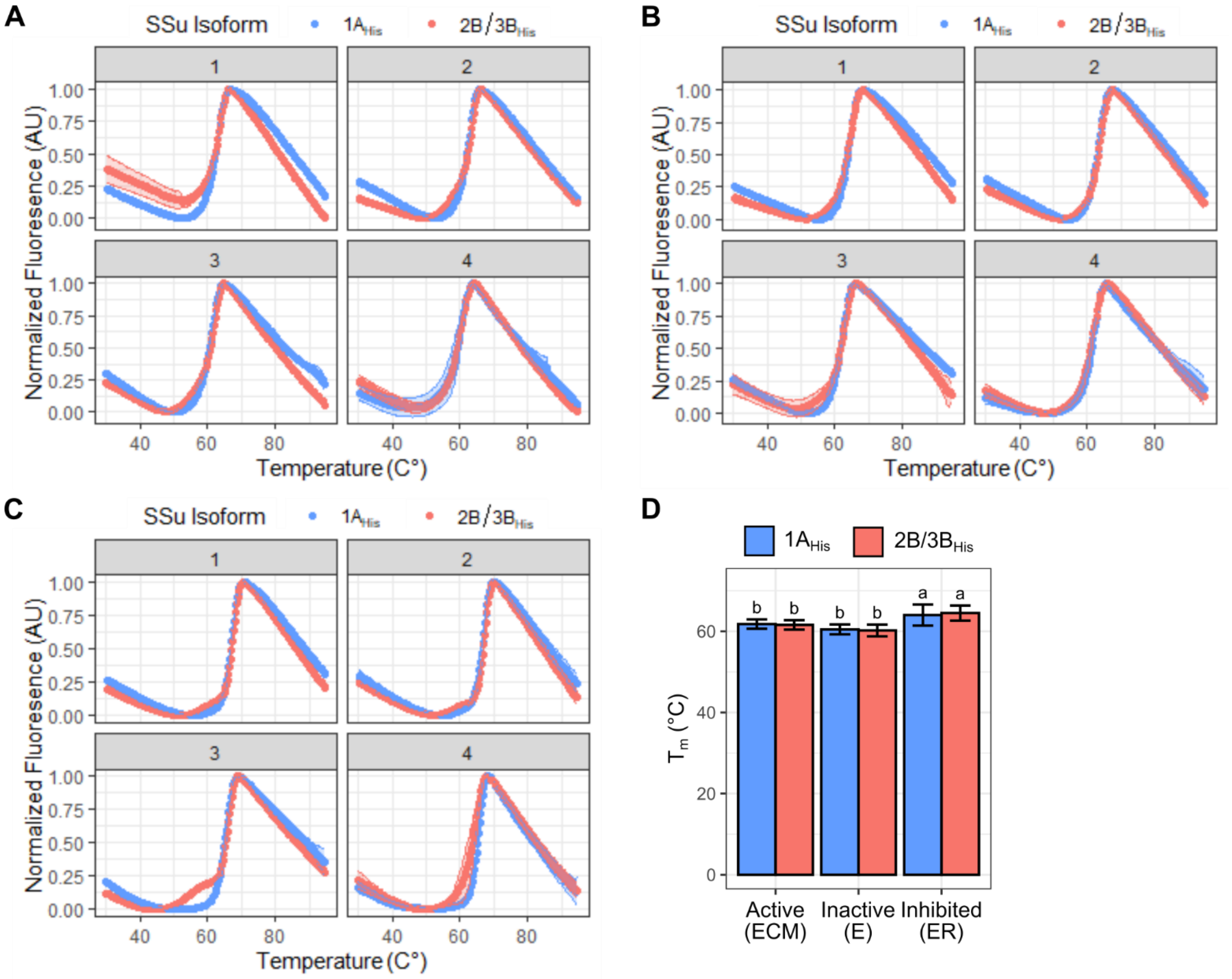

Fig. S12: Melting temperature curves from thermal shift assay

Melting temperature curves for (A) inactive, (B) active, and (C) RuBP-inhibited Rubisco. Each panel represents a biological replicate, showing the mean of four technical replicates with standard deviation in shaded regions. (D) Mean melting temperature derived from melting temperature curves (n=16). Error bars indicate standard deviation. Letters indicate ranking of means following one-way ANOVA and post-hoc Tukey HSD test with significance cutoff 0.05.

Supplementary Tables

Table S1: Primers used for genotyping of *A. thaliana* T2 plants

Primers used to confirm transgene presence and identity in T2 plants. Primer sets A, B, and C uniquely identify each of the 4 pairwise combinations of SSus, and primer set D amplifies *ACT2* (AT3G18780) as a positive control (Fig. S2).

ID	Set	Binding site (strand)	Sequence
1	A	rbcS2B intron 2 (+)	CCTTTGTCTCTAAGCATCCTTTATTCTTCTTCTTCA TTGC
2	A,B	StrepTag (-)	CTTACTTTTCAAACTGCGGGTGTGACCA
3	B,C	HisTag (+)	CACCATCATCACCATCACTAAGCTTATATGAAGAT GAAG
4	C	rbcS1A intron 2 (-)	CCACATGTTACAAACCACTCAGATAACATTTTGAA CATG
5	D	ACT2 (+)	ATTCTCCGTTTTGAATCTTCCTCAATCTCATCTTCT
6	D	ACT2 (+)	TGGAATGTGCTGAGGGAAGCAAG

Table S2: Native and denatured mass spectrometry instrument parameters
Q-Exactive UHMR Quadrupole-Orbitrap Mass Spectrometer (Thermo Fisher Scientific) instrument parameters applied during analysis of native and denatured Rubisco.

Parameter	Native	Denatured
m/z range	350-25000	350-25000
Resolution	6250	6250
In-source CID (V)	60	20
In-Source CE (eV)	100	40
Source temperature (°C)	300	200
Capillary voltage (kV)	1.7	1.7
Source DC offset (V)	21	21
Inject flatapole DC (V)	8	8
Inter flatapole lens (V)	5	5
Bent flatapole DC (V)	15	2
Transfer multipole DC (V)	1	1
C-Trap Entrance Lens Tune Offset (V)	1.8	1.8
Pressure (mbar)	8	5

Table S3: Theoretical and measured masses of homo- and heterogeneous Rubisco
Theoretical masses were calculated by summing molecular weights of constituent subunits for $1A_{Strep}$, $2B/3B_{GB1\text{-}His}$, and $2B/3B_{His}$-$1A_{Strep}$ Rubiscos. Measured masses were determined via native MS.

		L_8S_8 mass (kDa)		
**# $1A_{Strep}$**	**# $2B/3B_{GB1\text{-}His}$**	**Theoretical**	**Measured**	**Error (kDa)**
8	0	549.55	548.08	1.47
7	1	556.26	-	
6	2	562.97	561.63	1.34
5	3	569.68	568.39	1.29
4	4	576.38	575.16	1.22
3	5	583.09	581.91	1.18
2	6	589.80	-	
1	7	596.51	-	
0	8	603.22	602.09	1.13

Table S4: V_{Cmax} and K_m^{RuBP} of homo- and heterogeneous Rubisco

Maximum carboxylation rate (V_{Cmax}) and substrate affinity (K_m^{RuBP}) measured at 25°C and 35°C for SSu-heterogeneous 2B/3B$_{His}$-1A$_{Strep}$ Rubisco and its pure counterparts. Values expressed as mean ± standard deviation from 5 technical replicates. Letters indicate ranking of means following one-way ANOVA and post-hoc Tukey HSD test with significance cutoff 0.05. A separate ANOVA and post-hoc Tukey HSD test was conducted per measurement temperature.

	Rubisco	V_{Cmax} (s^{-1})	K_m^{RuBP} (µM)
35°C	2B/3B$_{His}$	2.887 ± 0.076^{a}	84.56 ± 6.57^{a}
	1A$_{Strep}$	3.095 ± 0.075^{b}	106.39 ± 9.30^{b}
	2B/3B$_{His}$-1A$_{Strep}$	2.974 ± 0.062^{ab}	137.10 ± 13.34^{c}
25°C	2B/3B$_{His}$	1.173 ± 0.023^{a}	45.28 ± 14.07^{a}
	1A$_{Strep}$	1.364 ± 0.029^{b}	76.03 ± 12.91^{b}
	2B/3B$_{His}$-1A$_{Strep}$	1.272 ± 0.061^{c}	79.52 ± 12.68^{b}

Table S5: Biological replicates of VC for homo- and heterogeneous Rubisco
Spectrophotometric assay-measured carboxylation rate (V_C) at 25°C with 1000 µM RuBP for SSu-heterogeneous 2B/3B$_{His}$-1A$_{Strep}$ Rubisco and its pure counterparts. Each group of three rows contains data from a different biological replicate, representing a separate Rubisco purification and preparation of the assay enzyme mixture. Values expressed as mean ± standard deviation from 5 technical replicates. Letters indicate ranking of means following one-way ANOVA and post-hoc Tukey HSD test with significance cutoff 0.05. A separate ANOVA and post-hoc Tukey HSD test was conducted per biological replicate.

	**Rubisco**	**V_C (s^{-1})**
**Bio rep 1**	2B/3B$_{His}$	1.104 ± 0.063^{a}
	1A$_{Strep}$	1.270 ± 0.035^{b}
	2B/3B$_{His}$-1A$_{Strep}$	1.117 ± 0.070^{a}
**Bio rep 2**	2B/3B$_{His}$	1.111 ± 0.045^{a}
	1A$_{Strep}$	1.208 ± 0.031^{b}
	2B/3B$_{His}$-1A$_{Strep}$	1.190 ± 0.080^{ab}
**Bio rep 3**	2B/3B$_{His}$	1.095 ± 0.048^{a}
	1A$_{Strep}$	1.222 ± 0.068^{b}
	2B/3B$_{His}$-1A$_{Strep}$	1.169 ± 0.047^{ab}

Table S6: E_a for V_{Cmax} of homo- and heterogeneous Rubisco
Activation energy of max carboxylation rate (E_a for V_{Cmax}) of SSu-heterogeneous 2B/3B$_{His}$-1A$_{Strep}$ Rubisco and its pure counterparts, as calculated based on difference between V_{Cmax} at 25°C and 35°C. Values expressed as mean ± standard deviation from 5 technical replicates. Letters indicate ranking of means following one-way ANOVA and post-hoc Tukey HSD test with significance cutoff 0.05.

**Rubisco**	**E_a for V_{Cmax} (kJ)**
2B/3B$_{His}$	68.601 ± 1.219[a]
1A$_{Strep}$	62.541 ± 0.882[b]
2B/3B$_{His}$-1A$_{Strep}$	64.886 ± 3.679[ab]

Table S7: v_i, v_f, and k_{obs} of homo- and heterogeneous Rubisco
Initial velocity (v_i), final velocity (v_f), and first order rate constant of inhibitor release (k_{obs}) measured at 25°C for RuBP-inhibited SSu-heterogeneous 2B/3B$_{His}$-1A$_{Strep}$ Rubisco and its pure counterparts. Values expressed as mean ± standard deviation from 5 technical replicates. Letters indicate ranking of means following one-way ANOVA and post-hoc Tukey HSD test with significance cutoff 0.05.

**Rubisco**	**v_i (s^{-1})**	**v_f (s^{-1})**	**k_{obs} (ms^{-1})**
2B/3B$_{His}$	0.014 ± 0.003[ab]	0.411 ± 0.062[ab]	1.391 ± 0.275[a]
1A$_{Strep}$	0.020 ± 0.003[a]	0.500 ± 0.091[a]	1.399 ± 0.246[a]
2B/3B$_{His}$-1A$_{Strep}$	0.001 ± 0.003[b]	0.363 ± 0.028[b]	2.038 ± 0.244[b]

Table S8: Cryo-EM data collection and refinement parameters
Overview of parameters applied during cryo-EM data collection and refinement for $1A_{His}$ and $2B/3B_{His}$ structures.

	**$1A_{His}$**	**$2B/3B_{His}$**
Mg^{2+}	+	-
PDB	9N37	9MUR
EMBD	EMD-48847	EMD-48648
**Data collection**		
EM equipment	Titan Krios	Talos Arctica
Voltage (kV)	300	200
Detector	K3	K3
Frames	40	40
Defocus range (μm)	-0.6 to -2.4	-0.5 to -2.5
Electron dose (e-/Å^2)	40	50
Magnification (X)	130000	79000
Pixel size (Å)	0.664	1.07
Movies	2976	1229
Final Particles	256188	249734
Symmetry imposed	D4	D4
Map Resolution (Å)	2.21	2.48
**Model Refinement**		
	R.M.S. deviation	
Bond lengths (Å)	0.004	0.006
Bond angles (°)	0.611	0.747
	Validation score	
Molprobity score	1.32	2.96
Clashscore	2.09	4.30
Poor rotamers (%)	1.29	3.51
CaBLAM outliers (%)	2.64	3.64
	Ramachandran plot	
Favored (%)	96.25	95.23
Allowed (%)	3.73	4.77
Outliers (%)	0.02	0.00

Table S9: Alignment statistics for superimposed $1A_{His}$ and $2B/3B_{His}$ structures
Alignment statistics from Gesamt in the CCP4 suite (26, 27) for superimposed $1A_{His}$ and $2B/3B_{His}$ structures.

Alignment	RMSD	Q score	Aligned residues	% identity
L_8S_8	0.438	0.574	3328	99.8%
L	0.391	0.947	416	99.8%
L_2	0.412	0.945	832	99.8%
S	0.499	0.933	117	94.9%
LS	0.422	0.943	533	98.7%
L_2S_2	0.420	0.575	832	99.8%

Table S10: Interface statistics for $1A_{His}$ and $2B/3B_{His}$ structures
Comparison of interfaces between $1A_{His}$ and $2B/3B_{His}$ structures. Values for $1A_{His}$ and $2B/3B_{His}$ reported as mean ± standard deviation. Number of replicates corresponds to the number of interfaces in the L_8S_8 holoenzyme: LL1, n=4; LL2, n=4; LL3, n=8; LS1, n=8; LS2, n=8; SS1, n=8. Values for $1A_{His}$:$2B/3B_{His}$ are expressed as the ratio of mean values of $1A_{His}$:$2B/3B_{His}$. Data obtained from PDBsum (28).

	$1A_{His}$			
**Interface**	**Area (Å^2)**	**# of residues**	**Hydrogen bonds**	**Nonbonded contacts**
LL1	5361.5 ± 13.6	102.8 ± 0.8	25.5 ± 2.1	225.5 ± 4.8
LL2	819.8 ± 7.9	16 ± 0	2 ± 0	17 ± 0
LL3	945.3 ± 3.7	14.5 ± 0.5	1 ± 0	20.8 ± 0.7
LS1	3262.1 ± 9	55.9 ± 0.9	7.9 ± 0.3	114.9 ± 1.8
LS2	1764.4 ± 15.2	26.3 ± 0.8	6.6 ± 0.7	56.5 ± 1
SS1	497.9 ± 5.4	8.1 ± 0.3	0 ± 0	9.1 ± 1.1
	$2B/3B_{His}$			
**Interface**	**Area (Å^2)**	**# of residues**	**Hydrogen bonds**	**Nonbonded contacts**
LL1	5563.8 ± 25.5	107.5 ± 0.5	20.5 ± 0.5	240.8 ± 4
LL2	870.3 ± 1.8	14 ± 0	2 ± 0	21 ± 0
LL3	927.5 ± 4.6	18.8 ± 0.7	2.1 ± 0.3	19.1 ± 1.7
LS1	3549.8 ± 8.6	60.6 ± 1.1	10 ± 0.5	132.3 ± 2.2
LS2	1745.1 ± 12.3	32.6 ± 1	3.6 ± 0.5	52.1 ± 2.2
SS1	614.8 ± 1.1	9.5 ± 0.9	0 ± 0	8.4 ± 0.7
	Ratio $1A_{His}$:$2B/3B_{His}$			
**Interface**	**Area (Å^2)**	**# of residues**	**Hydrogen bonds**	**Nonbonded contacts**
LL1	0.964	0.956	1.24	0.937
LL2	0.942	1.14	1.0	0.810
LL3	1.02	0.773	0.471	1.08
LS1	0.919	0.922	0.788	0.869
LS2	1.01	0.805	1.83	1.08
SS1	0.810	0.855	n/a	1.09

Table S11: Thermal shift assay-derived thermodynamic values
Thermodynamic values obtained for Rubisco containing the $1A_{His}$ or $2B/3B_{His}$ SSu isoform calculated from thermal shift assay. Values expressed as mean ± standard deviation from 16 replicates (4 biological x 4 technical replicates).

	**$1A_{His}$**	**$2B/3B_{His}$**
**Active (ECM)**		
T_m (°C)	61.05±0.4	60.55±0.54
$\Delta G°_{unfolding}$ (kJ/mol)	72.85±2.04	42.2±1.03
$\Delta S°_{unfolding}$ (kJ/(mol·K))	2.01±0.04	1.18±0.04
$\Delta H°_{unfolding}$ (kJ/mol)	672.55±15.18	394.56±12.1
**Inactive (E)**		
T_m (°C)	59.74±0.33	59.41±0.12
$\Delta G°_{unfolding}$ (kJ/mol)	60.35±1.98	48.06±0.63
$\Delta S°_{unfolding}$ (kJ/(mol·K))	1.73±0.04	1.39±0.01
$\Delta H°_{unfolding}$ (kJ/mol)	575.81±14.55	462.49±4.77
**Inhibited (ER)**		
T_m (°C)	64.01±2.59	65.03±1.87
$\Delta G°_{unfolding}$ (kJ/mol)	66.07±8.9	49.45±3.19
$\Delta S°_{unfolding}$ (kJ/(mol·K))	1.68±0.13	1.24±0.13
$\Delta H°_{unfolding}$ (kJ/mol)	566.66±47.03	417.59±41.55

Table S12: Interface statistics for PDB 1AUS and PDB 3RUB
Comparison of LL1 and LS1 interfaces between activated, unliganded *Spinacia oleracea* Rubisco (PDB 1AUS) and unactivated, unliganded *Nicotiana tabacum* Rubisco (PDB 3RUB) (33, 34). Number of replicates equals 1 for all interfaces because both 1AUS and 3RUB are crystal structures. Data obtained from PDBsum (28). See Fig. S11 for interface definitions.

	PDB 1AUS			
**Interface**	**Area (Å^2)**	**# of residues**	**Hydrogen bonds**	**Nonbonded contacts**
LL1	6940	146	46	405
LS1	3675	75	16	212
	**PDB 3RUB**			
**Interface**	**Area (Å^2)**	**# of residues**	**Hydrogen bonds**	**Nonbonded contacts**
LL1	6436	138	50	409
LS1	3612	69	16	218
	**Ratio 1AUS:3RUB**			
**Interface**	**Area (Å^2)**	**# of residues**	**Hydrogen bonds**	**Nonbonded contacts**
LL1	1.07	1.05	0.92	0.99
LS1	1.01	1.08	1	0.97

Legends for Supplementary Datasets

Dataset S1 (separate file): Metadata for all Form I Rubisco structures uploaded to PDB
List contains all structures available as of 2025 April 10 and is sorted by LSu sequence similarity to *A. thaliana*.

Dataset S2 (separate file): Plasmids used in this study

Dataset S3 (separate file): Western blotting data used for quantification of His-skew and heterogeneity
Western blotting data from dual SSu purifications and associated single SSu dilution series.

Dataset S4 (separate file): Raw data from kinetic assays
Spectrophotometer-measured change in $A_{340\ nm}$ over 120 s for ECM Rubisco, or 600 s for ER Rubisco.

SI References

1. C. Engler, R. Kandzia, S. Marillonnet, A One Pot, One Step, Precision Cloning Method with High Throughput Capability. *PLoS ONE* **3**, e3647 (2008).

2. E. Weber, C. Engler, R. Gruetzner, S. Werner, S. Marillonnet, A modular cloning system for standardized assembly of multigene constructs. *PloS One* **6**, e16765 (2011).

3. C. Engler, *et al.*, A golden gate modular cloning toolbox for plants. *ACS Synth. Biol.* **3**, 839–843 (2014).

4. T. L. Shimada, T. Shimada, I. Hara-Nishimura, A rapid and non-destructive screenable marker, FAST, for identifying transformed seeds of Arabidopsis thaliana. *Plant J.* **61**, 519–528 (2010).

5. N. Atkinson, Y. Mao, K. X. Chan, A. J. McCormick, Condensation of Rubisco into a proto-pyrenoid in higher plant chloroplasts. *Nat. Commun.* **11**, 6303 (2020).

6. J. Ng, Z. Guo, O. Mueller-Cajar, Rubisco activase requires residues in the large subunit N terminus to remodel inhibited plant Rubisco. *J. Biol. Chem.* **295**, 16427–16435 (2020).

7. S. J. Clough, A. F. Bent, Floral dip: a simplified method for Agrobacterium-mediated transformation of Arabidopsis thaliana. *Plant J.* **16**, 735–743 (1998).

8. J. Li, J. Chory, “Preparation of DNA from Arabidopsis” in *Arabidopsis Protocols*, J. M. Martinez-Zapater, J. Salinas, Eds. (Humana Press, 1998), pp. 55–60.

9. H. Aigner, *et al.*, Plant RuBisCo assembly in E. coli with five chloroplast chaperones including BSD2. *Science* **358**, 1272–1278 (2017).

10. R. H. Wilson, G. Thieulin-Pardo, F. Hartl, M. Hayer-Hartl, Improved recombinant expression and purification of functional plant Rubisco. *Febs Lett.* **593**, 611–621 (2019).

11. M. T. Lin, W. D. Stone, V. Chaudhari, M. R. Hanson, Small subunits can determine enzyme kinetics of tobacco Rubisco expressed in Escherichia coli. *Nat. Plants* **6**, 1289–1299 (2020).

12. J. Schindelin, *et al.*, Fiji: an open-source platform for biological-image analysis. *Nat. Methods* **9**, 676–682 (2012).

13. R Core Team, R: A language and environment for statistical computing. (2021). Deposited 2021.

14. A. Laganowsky, E. Reading, J. T. S. Hopper, C. V. Robinson, Mass spectrometry of intact membrane protein complexes. *Nat. Protoc.* **8**, 639–651 (2013).

15. M. T. Marty, *et al.*, Bayesian Deconvolution of Mass and Ion Mobility Spectra: From Binary Interactions to Polydisperse Ensembles. *Anal. Chem.* **87**, 4370–4376 (2015).

16. D. S. Kubien, C. M. Brown, H. J. Kane, “Quantifying the Amount and Activity of Rubisco in Leaves” in *Photosynthesis Research Protocols*, (Humana Press, Totowa, NJ, 2011), pp. 349–362.

17. Z. G. Oh, *et al.*, Unique biogenesis and kinetics of hornwort Rubiscos revealed by synthetic biology systems. *Mol. Plant* **17**, 1833–1849 (2024).

18. C. R. G. Sales, G. E. Degen, A. B. da Silva, E. Carmo-Silva, "Spectrophotometric Determination of RuBisCO Activity and Activation State in Leaf Extracts" in *Photosynthesis: Methods and Protocols*, S. Covshoff, Ed. (Springer, 2018), pp. 239–250.

19. F. G. Pearce, T. J. Andrews, The Relationship between Side Reactions and Slow Inhibition of Ribulose-bisphosphate Carboxylase Revealed by a Loop 6 Mutant of the Tobacco Enzyme*. *J. Biol. Chem.* **278**, 32526–32536 (2003).

20. A. Punjani, J. L. Rubinstein, D. J. Fleet, M. A. Brubaker, cryoSPARC: algorithms for rapid unsupervised cryo-EM structure determination. *Nat. Methods* **14**, 290–296 (2017).

21. A. Punjani, H. Zhang, D. J. Fleet, Non-uniform refinement: adaptive regularization improves single-particle cryo-EM reconstruction. *Nat. Methods* **17**, 1214–1221 (2020).

22. E. C. Meng, *et al.*, UCSF ChimeraX: Tools for structure building and analysis. *Protein Sci.* **32**, e4792 (2023).

23. P. Emsley, B. Lohkamp, W. G. Scott, K. Cowtan, Features and development of *Coot*. *Acta Crystallogr. D Biol. Crystallogr.* **66**, 486–501 (2010).

24. D. Liebschner, *et al.*, Macromolecular structure determination using X-rays, neutrons and electrons: recent developments in Phenix. *Acta Crystallogr. Sect. Struct. Biol.* **75**, 861–877 (2019).

25. J. Zivanov, T. Nakane, S. H. W. Scheres, Estimation of high-order aberrations and anisotropic magnification from cryo-EM data sets in RELION-3.1. *IUCrJ* **7**, 253–267 (2020).

26. E. Krissinel, Enhanced fold recognition using efficient short fragment clustering. *J. Mol. Biochem.* **1**, 76–85 (2012).

27. J. Agirre, *et al.*, The CCP4 suite: integrative software for macromolecular crystallography. *Acta Crystallogr. Sect. Struct. Biol.* **79**, 449–461 (2023).

28. R. A. Laskowski, J. Jabłońska, L. Pravda, R. S. Vařeková, J. M. Thornton, PDBsum: Structural summaries of PDB entries. *Protein Sci.* **27**, 129–134 (2018).

29. A. M. N. Joiner, J. C. Fromme, Structural basis for the initiation of COPII vesicle biogenesis. *Structure* **29**, 859-872.e6 (2021).

30. P. Di Tommaso, *et al.*, T-Coffee: a web server for the multiple sequence alignment of protein and RNA sequences using structural information and homology extension. *Nucleic Acids Res.* **39**, W13–W17 (2011).

31. X. Robert, P. Gouet, Deciphering key features in protein structures with the new ENDscript server. *Nucleic Acids Res.* **42**, W320–W324 (2014).

32. M. van Lun, J. S. Hub, D. van der Spoel, I. Andersson, CO2 and O2 Distribution in Rubisco Suggests the Small Subunit Functions as a CO2 Reservoir. *J. Am. Chem. Soc.* **136**, 3165–3171 (2014).

33. T. C. Taylor, I. Andersson, Structural transitions during activation and ligand binding in hexadecameric Rubisco inferred from the crystal structure of the activated unliganded spinach enzyme. *Nat. Struct. Biol.* **3**, 95–101 (1996).

34. P. M. Curmi, D. Cascio, R. M. Sweet, D. Eisenberg, H. Schreuder, Crystal structure of the unactivated form of ribulose-1,5-bisphosphate carboxylase/oxygenase from tobacco refined at 2.0-A resolution. *J. Biol. Chem.* **267**, 16980–16989 (1992).
